# Double Imprinted Nanoparticles for Sequential Membrane‐to‐Nuclear Drug Delivery

**DOI:** 10.1002/advs.202309976

**Published:** 2024-07-08

**Authors:** Pankaj Singla, Thomas Broughton, Mark V. Sullivan, Saweta Garg, Rolando Berlinguer‐Palmini, Priyanka Gupta, Katie J Smith, Ben Gardner, Francesco Canfarotta, Nicholas W. Turner, Eirini Velliou, Shoba Amarnath, Marloes Peeters

**Affiliations:** ^1^ Department of Chemical Engineering The University of Manchester Engineering building A, East Booth Street, Oxford Road Manchester M13 9PL UK; ^2^ School of Engineering Newcastle University Merz Court, Claremont Road Newcastle Upon Tyne NE1 7RU UK; ^3^ Center for Cancer Research, NU Cancer, Faculty of Medical Sciences Newcastle University Newcastle Upon Tyne NE2 4HH UK; ^4^ Immune Regulation Laboratory, NU Biosciences, Faculty of Medical Sciences Newcastle University Newcastle Upon Tyne NE2 4HH UK; ^5^ NIHR, Biomedical Research Centre Newcastle University Newcastle Upon Tyne NE2 4HH UK; ^6^ Department of Chemistry University of Sheffield Dainton Building Sheffield S3 7HF UK; ^7^ The Bio‐Imaging Unit, Medical School Newcastle University William Leech Building Newcastle Upon Tyne NE2 4HH UK; ^8^ Centre for 3D models of Health and Disease, Division of Surgery and Interventional Science University College London London W1W 7TY UK; ^9^ MIP Discovery The Exchange Building, Colworth Park, Sharnbrook Bedford MK44 1LQ UK

**Keywords:** biomimetic 3D cancer models, breast cancer, chemotherapy, imprinted nanoparticles, precision nanomedicine, targeted drug delivery

## Abstract

Efficient and site‐specific delivery of therapeutics drugs remains a critical challenge in cancer treatment. Traditional drug nanocarriers such as antibody‐drug conjugates are not generally accessible due to their high cost and can lead to serious side effects including life‐threatening allergic reactions. Here, these problems are overcome via the engineering of supramolecular agents that are manufactured with an innovative double imprinting approach. The developed molecularly imprinted nanoparticles (nanoMIPs) are targeted toward a linear epitope of estrogen receptor alfa (ERα) and loaded with the chemotherapeutic drug doxorubicin. These nanoMIPs are cost‐effective and rival the affinity of commercial antibodies for ERα. Upon specific binding of the materials to ERα, which is overexpressed in most breast cancers (BCs), nuclear drug delivery is achieved via receptor‐mediated endocytosis. Consequentially, significantly enhanced cytotoxicity is elicited in BC cell lines overexpressing ERα, paving the way for precision treatment of BC. Proof‐of‐concept for the clinical use of the nanoMIPs is provided by evaluating their drug efficacy in sophisticated three‐dimensional (3D) cancer models, which capture the complexity of the tumor microenvironment in vivo without requiring animal models. Thus, these findings highlight the potential of nanoMIPs as a promising class of novel drug compounds for use in cancer treatment.

## Graphical abstract

In advanced materials research, scientists aim to enhance chemotherapy, nucleic acid, and protein drug delivery using different materials, for instance, antibody‐drug conjugates, like Enhertu. These approaches can be inaccessible to many due to financial constraints or lack of expertise. This research focuses on a novel nanomaterial approach using double imprinting to improve targeted chemotherapy. These nanomaterials selectively target breast cancer cells with strong binding, low toxicity, and efficient drug release. A biomimetic scaffold‐assisted 3D model for breast cancer is developed. This cost‐effective technology can make advanced cancer treatments more accessible and affordable, potentially replacing traditional antibodies and peptides, and benefiting a wider community.

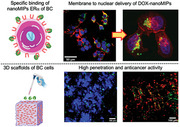

## 1 Introduction

Breast cancer (BC) is the most frequently diagnosed cancer worldwide; with 2.3 million new cases in 2020, this accounts for 1 in 8 cancer diagnoses.^[^ ^1^ ^]^ It is a highly heterogeneous disease that can be caused by a variety of distinct genetic alterations in mammary epithelial cells, requiring a combinatorial evaluation of the histopathology of the primary tumor and of the expression pattern of hormone receptors to determine the optimal patient treatment plan. The majority (70%) of BCs are estrogen receptor‐positive (ER+), meaning the cancer is fueled by the estrogen hormone.^[^ ^1^ ^]^ ERα is a central transcription factor that is often overexpressed in BC, but also in ovarian, endometrial, and prostate cancers,^[^ ^2^ ^]^ and plays a crucial role in breast tumorigenesis and proliferation of BC cells.

Traditionally, ERs have been considered nuclear receptors, but it has been well‐documented that these receptors are also present on the membrane and in the cytoplasm. Nuclear ERα stimulates gene expression changes that promote cell cycle progression.^[^ ^3^ ^]^ Moreover, the binding of ligands to plasma membrane ERα triggers rapid cellular changes through second messenger pathways, which also contribute to the transcriptional effects of estrogen by regulating processes such as proliferation, cell migration, and development.^[^ ^4^ ^]^ Membrane‐bound ERα receptors are associated with caveolae that help in their internalization through dynamin‐mediated processes and play a crucial role in initiating ERα signaling.^[^ ^5^ ^]^ Endocrine therapy, consisting of for instance tamoxifen and fulvestrant, is the preferred first line of treatment for ER‐positive BC.^[^ ^6^ ^]^ Chemotherapeutic drugs may also be prescribed alone or in combination with endocrine therapy.^[^ ^7^ ^]^ Additionally, chemotherapy may be used after surgical resection or in the case of metastatic BC, with doxorubicin (DOX) being one of the most frequently prescribed chemotherapy drugs for solid breast tumors. However, all current treatment options come with significant challenges and side effects, such as drug resistance, severe toxicity (for example, neurotoxicity and cardiac toxicity including irreversible heart injury), and allergic reactions (fainting, sweating) resulting from chemotherapy's non‐selective behavior.^[^ ^8^ ^]^

Nanoparticles are currently being utilized in the field of nanomedicine to enable targeted drug delivery of chemotherapeutic drugs, leading to more effective treatment of cancer while minimizing harmful side effects.^[^ ^9^ ^]^ To achieve targeted drug delivery, nanoparticles (e.g., gold nanoparticles, liposomes) are functionalized with targeting agents which can be broadly classified as proteins (antibodies and their fragments), nucleic acids (aptamers), or other receptor ligands (peptides, vitamins, and carbohydrates).^[^ ^10^ ^]^ Enhertu is a prime example of a commercial antibody‐drug conjugate for BC treatment: this product is composed of trastuzumab antibodies conjugated with deruxtecan and is designed to specifically target and treat metastatic HER2 (human epidermal growth factor receptor 2)‐positive BC. Another example is Nab‐paclitaxel (Abraxane), FDA‐approved albumin‐bound nanoparticles for the delivery of paclitaxel used as second‐line treatment for metastatic BC.^[^ ^11^ ^]^ However, these formulations all rely on biological counterparts, which may encounter significant limitations such as high cost (minimum ≈$100000 per treatment for Enhertu) limited in vivo stability, inherent heterogeneity of biologics which can lead to immune intolerance, and inability to recognize altered peptide antigens. These drawbacks are overcome using molecular imprinted polymeric nanoparticles (nanoMIPs), which are highly selective, cost‐effective, and robust, and can serve as an alternative for targeted drug delivery. These synthetic receptors are small porous polymeric nanostructures containing specific binding sites for a particular target.^[^ ^12^ ^]^ These materials possess several advantages such as excellent chemical and biological stability, biocompatibility, versatility, and fast binding kinetics.^[^ ^13^ ^]^ Solid‐phase synthesis for the manufacturing of nanoMIPs has led to the production of high‐affinity materials with homogeneous binding distribution and excellent biocompatibility due to the use of the solid phase as an affinity medium.^[^ ^14^ ^]^

There are some examples of hybrid core–shell systems where drug‐loaded nanoparticles are coated with MIPs to facilitate targeted drug delivery to cell‐specific receptors.^[^ ^15^ ^]^ However, a more scalable and straightforward approach would be to design and synthesize double imprinted nanoMIPs. In this work, we have developed DOX‐loaded nanoMIPs for their targeted delivery to ERα positive BC cell lines to improve breast cancer treatment via double imprinting with an epitope of ERα in the presence of DOX. Via this innovative synthesis approach, we have streamlined the manufacturing to a one‐stage process, which will significantly lower production cost and development time. There is one report on double imprinted nanoparticles where drug delivery of DOX is explored via targeting the membrane receptor epidermal growth factor receptor (EGFR). As this material is only bound to the receptor and was not internalized by the cells, the therapeutic effect in this case can be limited.^[^ ^12c^ ^]^ Our work introduces a novel approach by successfully targeting the membrane receptor ERα and facilitating the translocation of nanoMIPs toward the nucleus, which paves the way for the selective drug delivery of traditionally “undruggable” targets with MIPs.

In this context, nanoMIPs have been developed to target ERα and were tested on two different BC cell lines: MCF‐7 (ERα positive) and MDA‐MB‐231 (ERα negative), which represent different subtypes of BC and thus require different treatment in clinical practice. The study showed that nanoMIPs designed for ERα successfully bound to this receptor on the membrane and were subsequently internalized, thus facilitating highly specific intracellular delivery of DOX. By enabling nuclear delivery of DOX, the nanoMIPs elicited significantly higher cytotoxicity (80%) toward the MCF‐7 cell line as compared to the negative control cell line MDA‐MB‐231 (15%).

Furthermore, the efficacy was evaluated in a scaffold‐assisted 3D model of BC cell lines, which provides a better representation of BC progression and response to drugs in vivo. 3D cancer models provide a remarkable platform for in vitro testing of these nanoparticle formulations by effectively mimicking the complex tumor microenvironment found in living organisms.

There are regulatory demands to move away from traditional animal‐based safety assessment studies, and pharmaceutical industries are increasingly interested in alternative methodologies to efficiently screen and characterize therapeutic molecules in the drug development pipeline.^[^ ^16^ ^]^ In addition, there is no animal model that can fully replicate processes within the human body. In the search for alternatives, microphysiological systems, 3D spheroids, organoids, and scaffolds are being used at singular and multicellular levels to screen drugs.^[^ ^17^ ^]^ 3D cancer models present a significant advancement over traditional 2D models in the study of human diseases.^[^ ^18^ ^]^ They more accurately replicate various features of the tissue microenvironment, including structural integrity, biochemical composition (mimicking the extracellular matrix), and cellular diversity, offering a robust and personalized approach. This level of detail and specificity renders 3D cancer models superior to animal models, as they enable the creation of patient‐specific tissue models in vitro.^[^ ^18^ ^]^ Furthermore, 3D cancer models are excellent surrogates for studying tissue penetration, especially when their mechanical properties and cell density closely mimic those of actual human tissues.^[^ ^18^, ^19^ ^]^ However, 3D cancer models may not adequately represent the processes of absorption, distribution, metabolism, and elimination (ADME). Therefore, microphysiological systems are continually being explored, utilizing multiple organ models to address these limitations and enable effective pharmaceutical testing without the use of animals.^[^ ^20^ ^]^

This work builds upon the development of engineered nanoMIPs as a targeted drug delivery vehicle in the field of precision medicine, adhering to the 3Rs (replace, reduce, and refine) principles of animal research. This approach can easily be expanded to other cancer types, given the versatility of the method used to manufacture these engineered nanoparticles.

## 2 Results and Discussions

### 2.1 Solid Phase Synthesis and Characterization of NanoMIPs

**Figure 1:**
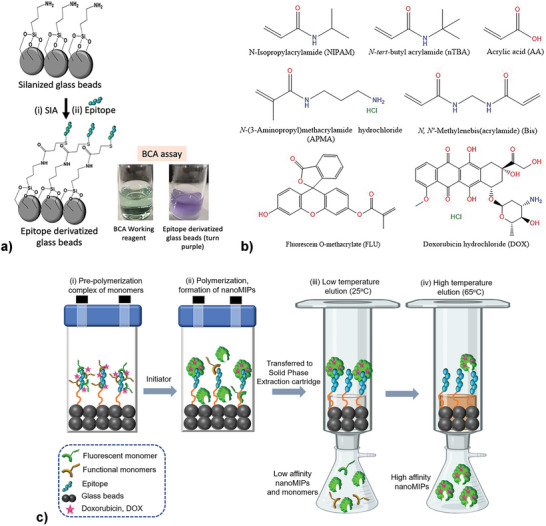
a) Molecular structures of different functional monomers, fluorescent monomer (Fluorescein‐o‐methacrylate), and DOX b) Schematic of conjugation of cysteine modified epitope with silanized glass beads and confirmation through bicinchoninic acid (BCA) assay (green to purple in the presence of epitope modified glass beads) c) Schematic of synthesis of double imprinted nanoMIPs: the primary template is the epitope conjugated on silanized beads, whilst DOX acts as secondary template in solution.

We have produced nanoMIPs to target the human ERα receptor protein (595 amino acids) with a molecular weight of 66 kDa (ERα66 wild type). A linear epitope sequence SHSLQKYYITGEAEGFPATV (576‐595 amino acids at the C‐terminus, corresponding to the binding region of anti‐ERα Antibody F‐10, sc‐800, Santa Cruz Biotechnology, Inc. USA) on helix 12 of human ERα receptor (UniProtKB‐P03372) was selected.^[^ ^21^ ^]^ A cysteine residue was added to the N‐terminus of this peptide sequence for binding to the solid support used to produce the nanoMIPs, and its attachment was confirmed via a BCA assay (**Figure** **1**a). To circumvent the limitations connected with the use of whole proteins, the epitope imprinting approach offers several advantages viz. lower costs, compatibility with different synthetic conditions (pH, temperatures, and solvents), selectivity toward the target, greater versatility, no need for costly and lengthy protein purification steps.^[^ ^22^ ^]^ The epitope‐imprinting method is well‐established and MIP‐based sensors produced with this approach have successfully achieved full protein identification.^[^ ^12^, ^23^ ^]^

A variety of monomers were employed, comprising *N*‐Isopropylacrylamide (NIPAM) for hydrogen bonding, *N*‐*tert*‐Butylacrylamide (nTBA) for hydrophobic interactions, and *N‐*(3‐Aminopropyl)methacrylamide hydrochloride (APMA), and acrylic acid (AA) for ionic interactions. Fluorescein‐o‐methacrylate was added to the monomer mixture to obtain fluorescent active nanoMIPs that enable tracking of the nanoparticles in the 2D cell lines and 3D cancer models when imaging of the system. The molecular structures of the monomers and DOX are depicted in Figure 1b, while the composition of various batches of nanoMIPs and NIPs produced in this study is presented in Table S1 (Supporting Information). Fluorescein‐tagged nanoMIPs without DOX (unloaded) are referred to as FLU‐nanoMIPs, whereas DOX loaded, and fluorescein tagged DOX loaded double imprinted nanoparticles are named DOX‐nanoMIPs and FLU‐DOX‐nanoMIPs respectively. Non‐imprinted nanoparticles (NIPs, named as FLU‐NIPs and FLU‐DOX‐NIPs were also prepared through a solid phase synthesis approach using silanized beads and serve as a reference. The solid‐phase synthesis method for the manufacturing of the different nanoMIPs is depicted in Figure 1c.

**Figure 2:**
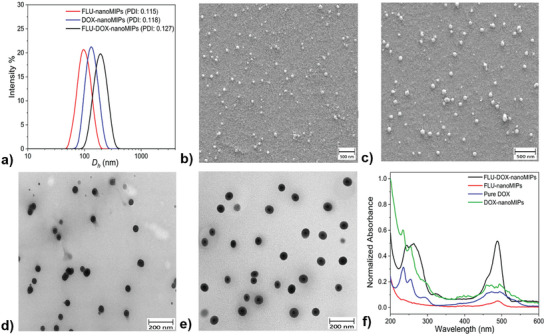
Characterization of different nanoMIPs a) Intensity weighted size distribution plot (DLS measurement) FLU‐nanoMIPs (*D_h_* = 121.16 ± 2.70), DOX‐nanoMIPs (*D_h_* = 141 ± 2.76) and FLU‐DOX‐nanoMIPs (*D_h_* = 168 ± 2.24) at 25 °C. The Z‐average *D_h_* of the nanoMIPs was determined using cumulant analysis by the equipment, and the standard deviation was calculated based on five measurements; representative SEM images of b) FLU‐nanoMIPs c) FLU‐DOX‐nanoMIPs; TEM image (25000×) of d) FLU nanoMIPs, e) FLU‐DOX‐nanoMIPs; f) UV‐spectra of Pure DOX, FLU‐nanoMIPs, DOX‐nanoMIPs and FLU‐DOX‐nanoMIPs.

The hydrodynamic diameter (*D_h_*) of FLU‐nanoMIPs, DOX‐nanoMIPs, and FLU‐DOX‐nanoMIPs were found to be 121 ± 3 nm (PDI = 0.115), 141 ± 3 nm (PDI = 0.118) and 168 ± 2 nm (PDI = 0.127) respectively (**Figure** **2**a). These results showed that the loading of DOX into nanoMIPs increased *D_h_* of nanoMIPs, due to accommodating fluorescein‐o‐methacrylate and DOX within the nanoMIPs. PDI values of these nanoMIPs were found to be less than 0.2 suggesting that these nanoparticles are homogeneous. Moreover, the *D_h_* of control nanoMIPs and different NIPs viz. control NIPs, FLU‐NIPs, and FLU‐DOX‐NIPs are shown in Table S2 (Supporting Information). The average size from the scanning electron microscopy (SEM) measurements for the FLU‐nanoMIPs and FLU‐DOX‐nanoMIPs were observed to be 46 ± 11 and 60 ± 10 nm (Figure 2b,c) respectively, whereas DOX‐nanoMIPs showed the size of 58 ± 6 (Figure S1a, Supporting Information). This was in line with the transmission electron microscopy (TEM) results, where the size of FLU‐nanoMIPs and FLU‐DOX‐nanoMIPs was found to be 40 ± 6 nm and 58 ± 6 respectively (Figure 2d,e) and DOX‐nanoMIPs showed size of 57 ± 0.9 nm (Figure S1b, Supporting Information). Moreover, both methodologies confirmed that the nanoMIPs exhibited a spherical morphology. The larger size observed in DLS measurements can be attributed to the swelling of the nanoMIPs, primarily induced by the copolymers of AA and NIPAM monomers, which are highly prone to swelling in the liquid state upon water absorption.^[^ ^24^ ^]^ In contrast, the sizes obtained from SEM and TEM reflect the nanoMIPs' dimensions in the dry state, where no water is available to facilitate swelling, and the nanoMIPs exist in a collapsed state.^[^ ^25^ ^]^ Another factor contributing to the discrepancy between the sizes obtained from SEM/TEM and DLS is the potential presence of certain oligomers that adhere to the monodispersed nanoparticles, resulting in an increase in the hydrodynamic diameter (*D_h_*).^[^ ^26^ ^]^ This phenomenon can lead to a larger apparent size when measured through DLS. The variation in sizes observed between nanoMIPs and DOX‐loaded nanoMIPs was primarily attributed to the incorporation of DOX into the nanoMIPs, resulting in an overall increase in their size.

UV‐spectres of different batches DOX loaded nanoMIPs have been shown in Figure 2f and estimation of DOX loading was performed by determining the amount of imprinted drug using a calibration curve (Figure S2, Supporting Information), as well as assessing the loading efficiency and loading capacity (Table S3, Supporting Information). The loading of DOX into DOX‐nanoMIPs, FLU‐DOX‐nanoMIPs, and FLU‐DOX‐NIPs was found to be 17.28 ± 0.1, 19.33 ± 0.16, and 18.37 ± 0.12 µg/100 µg of nanoMIPs, respectively, with corresponding drug loading efficiencies of 57.6 ± 0.33%, 64.43 ± 0.53%, and 61.12 ± 0.40%. This DOX loading is comparable to the Doxosome (DOX 20 µg/ ≈160 µg of lipids), which is a commercially available liposomal nanoformulation specifically designed for research and development purposes.^[^ ^27^ ^]^

#### 2.1.1 Time‐Dependent Size Distribution of NanoMIPs in Cell Culture Media

To understand the size distribution and stability of nanoMIPs in culture media, DLS measurements have been carried out at different time incubations (0, 0.5, 4, 8, 12, and 24 h) at 37 °C. *D_h_* of various nanoMIPs in DMEM culture media at different time intervals is shown in Figure S3 (Supporting Information). The size of the nanoMIPs did not vary between incubation points, which indicated that these nanoMIPs are stable and its spherical morphology remained intact. Meesaragandla and colleagues also reported similar results, where they demonstrated the stability of dextrin‐coated gold nanoparticles in protein‐rich culture media.^[^ ^28^ ^]^ However, there was a small increase in *D_h_* of nanoMIPs observed which might be due to the interactions of biomolecules in culture media and nanoMIPs. In addition to that, results from in vitro tests such as cytotoxicity, flow‐cytometry, and confocal microscopy showed that these nanoMIPs remained stable in the complex culture media and retained binding affinity. Overall, the results evidenced the stability of the nanoMIPs in culture media.

#### 2.1.2 DOX Release Analysis

In this study, DOX release from different imprinted and non‐imprinted polymeric nanoparticles, viz. FLU‐DOX‐nanoMIPs, FLU‐DOX‐nanoNIPs, and DOX‐nanoMIPs, have been assessed at physiological pH (PBS solution, pH 7.4). All the nanoMIPs and nanoNIPs under investigation showed controlled release of DOX and a similar pattern of drug release because the monomer composition is similar, except for DOX‐nanoMIPs, where fluorescein‐o‐methacrylate is absent (Figure S4, Supporting Information). Complete drug release was shown in both the FLU‐DOX‐nanoMIPs and FLU‐DOX‐nanoNIPs in 72 h, whereas DOX‐nanoMIPs showed complete DOX release in 68 h. The controlled release of DOX is ascribed to the protective effect of the MIP layer around DOX since the drug is released via a diffusion mechanism. These nanoMIPs showed a pattern of sustained release of DOX, which not only prevents burst release of the drug which can lead to side‐effects but also helps to maintain DOX at a consistent level to enhance its therapeutic effect.

### 2.2 Binding Performance and Selectivity of NanoMIPs

**Figure 3:**
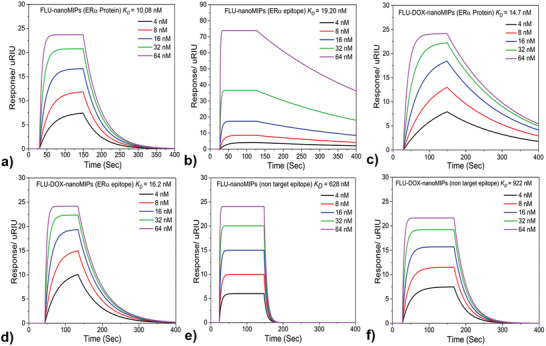
Typical sensorgrams depicting binding of different nanoMIPs a) FLU‐nanoMIPs with ERα protein, b) FLU‐nanoMIPs with ERα epitope, c) FLU‐DOX‐nanoMIPs with ERα protein, d) FLU‐DOX‐nanoMIPs with ERα epitope, e) FLU‐nanoMIPs with non‐target epitope, and f) FLU‐DOX‐nanoMIPs with non‐target epitope.

The prepared nanoMIPs were tested for their binding affinity toward the ERα protein (68 kDa) and the ERα template epitope used in the imprinting process via surface plasmon resonance (SPR) in a solution of PBS pH 7.4 and 0.01% Tween 20. To compare the effect of DOX loading on nanoMIPs, two different types of nanoMIPs (FLU‐nanoMIPs and DOX‐FLU‐nanoMIPs) were explored for the affinity against both ERα protein and ERα epitope (CSHSLQKYYITGEAEGFPATV). The nanoMIPs were immobilized onto carboxymethyl dextran hydrogel‐coated gold (Au) chips through EDC‐NHS chemistry, where the amine moiety of nanoMIPs was crosslinked with the carboxymethyl dextran on the Au chip.^[^ ^29^ ^]^ The sensograms depicted in **Figure** **3**a, correspond to the association and dissociation between the ERα protein to FLU‐nanoMIPs, with a similar trend observed for the other set of experiments for FLU‐nanoMIPs with template epitope (Figure 3b), FLU‐DOX‐nanoMIPs with ERα protein and template epitope (Figure 3c,d) respectively. The increase in signal referred to the association of epitope/ERα protein to the nanoMIPs and the association constant (*K_on_*) has been determined. The decrease in the signal showed the dissociation phase by which the dissociation constant was calculated (*K_off_*). The binding affinity of a molecular interaction was quantified by the equilibrium dissociation constant (*K_D_*) calculated from the values of *K_off_ /K_on_*. Response increases linearly in the case of template epitope/ERα protein (from a concentration range of 4 to 64 nm) for both FLU‐nanoMIPs and FLU‐DOX‐nanoMIPs. The *K_D_* values for the entire ERα protein were found to be 10.8 and 14.7 nm for FLU‐nanoMIPs and FLU‐DOX‐nanoMIPs, respectively. In the case of template epitope, *K_D_* values came out to be 19.2 and 16.2 nm for FLU‐nanoMIPs and FLU‐DOX‐nanoMIPs respectively.

Typically, antibodies have *K_D_* values falling within the low micromolar range to nanomolar range, indicating moderate to high affinity for the antigens they bind to. However, antibodies that exhibit even stronger binding affinity are considered to be high‐affinity antibodies, with *K_D_* values falling within the low nanomolar to sub‐nanomolar range.^[^ ^30^ ^]^ Our results revealed that the binding affinity of our nanoMIPs was comparable to that of antibodies. Selectivity is a crucial factor in the development of nanoMIPs as a targeted drug delivery system because it determines the ability of nanoMIPs to selectively bind and deliver drugs to the desired target, such as cancer cells while minimizing their toxicity to healthy tissue.^[^ ^31^ ^]^ The selectivity of nanoMIPs was evaluated by using the nontarget epitope SSERIDKQIRYILDGISALR (epitope of interleukin 6), which has a similar molecular weight (M_w_) of 2333.64 Da. The results showed that the *K_D_* values for the nontarget epitope were ≈62 times higher for both FLU‐nanoMIPs and FLU‐DOX‐nanoMIPs as compared to the target ERα protein (Figure 3e,f). Specifically, results found that a 62‐fold higher concentration of the nontarget is required to occupy 50% of the nanoMIPs. These results demonstrated the high selectivity of the nanoMIPs for the target ERα protein, which is critical for their effectiveness as a targeted drug delivery system.

### 2.3 In Vitro Cell Binding and Specificity of NanoMIPs

**Figure 4:**
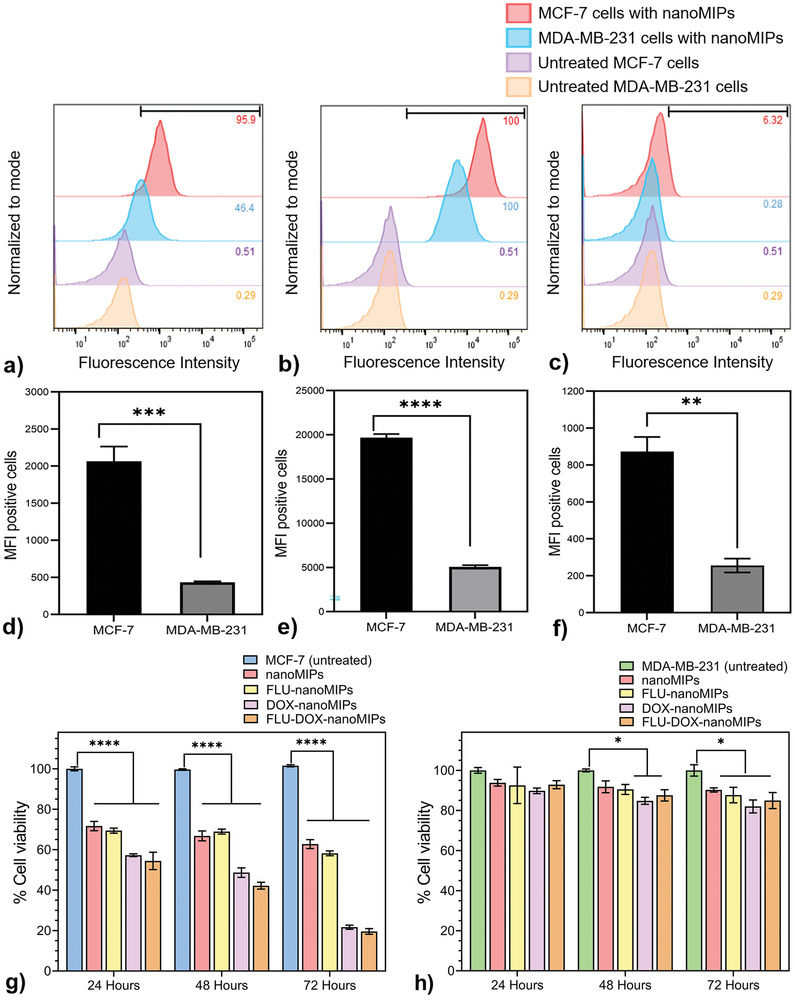
In vitro flow cytometry binding assay, cells were incubated with 10 µg mL^−1^ of a) FLU‐nanoMIPs, b) FLU‐DOX‐nanoMIPs, c) FLU‐DOX‐nanoMIPs; Mean Fluorescein intensity (MFI) positive cells (MCF‐7 vs MDA‐MB‐231) with binding of d) FLU‐nanoMIPs, e) FLU‐DOX‐nanoMIPs, f) DOX‐nanoMIPs. In vitro cell viability assay, at 10 µg mL^−1^ for each treatment of nanoMIPs, FLU‐nanoMIPs, DOX‐nanoMIPs, and FLU‐DOX‐nanoMIPs in g) MCF‐7 and h) MDA‐MB‐231. Data is expressed as mean ± standard error of the mean (SEM) of three measurements. d–f) ** *p* ≤ 0.01, *** *p* ≤ 0.001, **** *p* ≤ 0.0001 versus MCF‐7. g) **** *p* ≤ 0.0001 versus MCF‐7, h) * *p* ≤ 0.05 versus MDA‐MB‐231.

Two different BC cell lines MCF‐7 (ERα positive) and MDA‐MB‐231 (ERα negative) have been chosen to visualize the specific binding of nanoMIPs using flow cytometry. Flow cytometry binding for FLU‐nanoMIPs, FLU‐DOX‐nanoMIPs, and DOX‐nanoMIPs at 10 µg mL^−1^ concentration with MCF‐7 and MDA‐MB‐231 are represented in **Figure** **4**a,b,c respectively. These results demonstrate that there was significantly higher binding of the nanoMIPs (10 µg mL^−1^) to MCF‐7 cells in comparison to MDA‐MB‐231 cells. The higher the fluorescence intensity, the higher the binding of the nanoMIPs toward the cells overexpressing ERα was. The mean fluorescence intensity (MFI) plots of the FLU‐nanoMIPs (*p* ≤ 0.001), the FLU‐DOX‐nanoMIPs (*p* ≤ 0.0001), and the DOX‐nanoMIPs (*p* ≤ 0.01) shown in Figure 4d–f demonstrated that MCF‐7 cells exhibited approximately four‐folds greater number of MFI positive cells than MDA‐MB‐231 cells. However, low levels of binding with MDA‐MB‐231 cells have also been encountered, which can occur since some reports suggest that there is a small amount of ERα present in this cell line,^[^ ^32^ ^]^ in addition to the fact that all nanoparticles have some unavoidable nonspecific binding to the surface of cells.

In addition to that, the binding of each nanoMIPs with MCF‐7 cells at a higher concentration, 40 µg mL^−1^ was also tested, and the comparison graph is shown in Figure S5 (Supporting Information). The binding was found to be similar for each nanoMIP type, regardless of concentration. This suggests that the binding is mostly specific, and the lower concentration (10 µg mL^−1^) of nanoMIPs was sufficient to occupy most of the ERα. The comparison between FLU‐DOX‐nanoMIPs and FLU‐nanoMIPs revealed that the double imprinting of DOX in these nanoMIPs did not have an adverse effect on their binding performance. In fact, the binding capability was observed to be improved in the double imprinted nanoMIPs.

### 2.4 In Vitro Cytotoxicity Assessment

In vitro, the cytotoxicity of the different nanoMIPs has been assessed using a MTT assay. After treating MCF‐7 cells with different ER‐α nanoMIPs (FLU‐nanoMIPs, FLU‐DOX‐nanoMIPs, and DOX‐nanoMIPs), an increase in cell cytotoxicity was observed in a time‐dependent manner after 24, 48, and 72 h of incubation. Specifically, nanoMIPs and FLU‐nanoMIPs demonstrated significant cytotoxicity (28.3 ± 2.3% and 30.6 ± 1.2% respectively) after 24 h incubation with MCF‐7 cells, with only a 9% and 11% increase in cytotoxicity (total 37.2 ± 2.2% and 41.8 ± 1.2%) respectively after 72 h. Conversely, FLU‐DOX‐nanoMIPs and DOX‐nanoMIPs exhibited significant cytotoxicity in MCF‐7 cells (*p* ≤ 0.0001) even after 24 h (42.8 ± 0.7% and 45.5 ± 3.0%). As FLU‐DOX‐nanoMIPs and DOX‐nanoMIPs contain an equal amount of drug, therefore show a similar cytotoxicity trend in the case of both nanoMIPs. Furthermore, after 72 h, treatment with FLU‐DOX‐nanoMIPs and DOX‐nanoMIPs resulted in 80.4 ± 0.99% and 78.3 ± 1.03% cell death, respectively (Figure 4g). Similar in vitro cytotoxicity was observed for both DOX‐nanoMIPs and FLU‐DOX‐nanoMIPs, which was expected since both nanoMIPs have the same concentration of the drug. The cytotoxicity of FLU‐nanoMIPs, FLU‐DOX‐nanoMIPs, and DOX‐nanoMIPs toward MDA‐MB‐231 cells was found to be only 12.3 ± 2.7%, 15.0 ± 2.8%, and 18 ± 2.2%, respectively (as illustrated in Figure 4h). Moreover, control nanoMIPs (without FLU and DOX) also induced some cytotoxicity (36 ± 2%) in MCF‐7 cells after 72 h, where no significant cytotoxicity (9.8 ± 0.6%) with these nanoMIPs was observed in MDA‐MB‐231 cells (Figure S6, Supporting Information). The MTT assay results indicated a marked difference in cytotoxicity between MCF‐7 (ERα positive) and MDA‐MB‐231 (ERα negative) cell lines, which could be attributed to the selective binding and cellular uptake behavior of these nanoMIPs toward ERα positive cells. The FLU‐nanoMIPs exhibited selective cytotoxicity, while FLU‐DOX‐nanoMIPs and DOX‐nanoMIPs showed enhanced cytotoxicity to MCF‐7 cells. FLU‐nanoMIPs also elicited some cytotoxicity to the MCF‐7 cells, this could be due to their specific binding to the helix 12 (H12) region of the ER receptor, which plays a crucial role in dimerization and transcriptional activation.^[^ ^33^ ^]^

We also examined the cytotoxicity of non‐imprinted polymeric nanoparticles (NIPs), unloaded and DOX‐loaded NIPs (FLU‐NIPs and FLU‐DOX‐NIPs) in MCF‐7 and MDA‐MB‐231 cells. Results showed that FLU‐NIPs were highly biocompatible as both cell lines demonstrated cell viability of >90% even after 72 h of exposure. However, treatment with FLU‐DOX‐NIPs resulted in some cytotoxicity to MCF‐7 and MDA‐MB‐231 cells (20 ± 0.73% and 25 ± 2.8% after 72 h respectively) which was likely due to some diffusion of DOX from the nanoparticles over time (as seen in Figure S6a,b, Supporting Information). In addition, incubation with DOX showed a significant decrease in cell viability in both cell lines, which was consistent with previous literature reports.^[^ ^34^ ^]^ Furthermore, the selected batches of nanoMIPs have been investigated with live/dead assays in 3D cancer models described in sub‐Section 2.6.

### 2.5 CLSM Imaging

**Figure 5:**
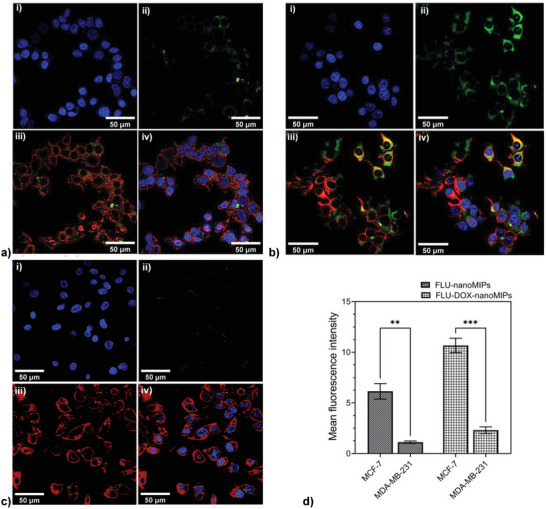
CLSM images (40×) of MCF‐7 cell line incubated for 1 h (at 37 °C) with a) FLU‐nanoMIPs and b) FLU‐DOX‐nanoMIPs; c) CLSM images (40×) for MDA‐MB‐231 incubated with FLU‐DOX‐nanoMIPs for 1 h at 37 °C i) DAPI, ii) FLU‐DOX‐nanoMIPs with green fluorescence, iii) plasma membrane with red fluorescence (WGA antibody Alexa Fluor™ 594) with FLU‐DOX‐nanoMIPs, iv) merged; d) The mean fluorescence intensity of FLU‐nanoMIPs and FLU‐DOX‐nanoMIPs in MCF‐7 cells and MDA‐MB‐231 cells. ** *p* ≤ 0.01, *** *p* ≤ 0.001, versus MCF‐7.

To assess the cellular uptake and internalization of nanoMIPs in BC cells, CLSM microscopy has been employed. MCF‐7 and MDA‐MB‐231 cells (≈80% confluency) were treated with FLU‐nanoMIPs and FLU‐DOX‐nanoMIPs (10 µg mL^−1^) in the chamber plates for 1 and 24 h at 37 °C. Moreover, a control experiment for ERα expression in both MCF‐7 and MDA‐MB‐231 was also performed and the results are depicted in Figure S7a,b (Supporting Information). The mean fluorescence intensity of ERα was observed to be significantly higher in MCF‐7 cells as compared to MDA‐MB‐231 cells (shown in Figure S7c, Supporting Information), indicating that the results are consistent with literature reports.^[^ ^35^ ^]^ Following the nanoMIPs incubation, the wells were washed with PBS (three times) to remove the nanoparticles that were not internalized or bound to the cells. DAPI and Alexa Fluor 594 wheat germ agglutinin (WGA) were used to stain the cell nucleus (blue) and plasma membrane (red) respectively. Following 1 h incubation, FLU‐nanoMIPs (**Figure** **5**a) and FLU‐DOX‐nanoMIPs (Figure 5b) were found to specifically bind with the plasma membrane of the MCF‐7 cells. On the other hand, minimal binding of FLU‐nanoMIPs (Figure S8, Supporting Information) and FLU‐DOX‐nanoMIPs (Figure 5c) with MDA‐MB‐231 cells were observed. Moreover, the difference in MFI (calculated from confocal images) between MCF‐7 cells and MDA‐MB‐231 cells treated with FLU‐nanoMIPs (*p* ≤ 0.01) and FLU‐DOX‐nanoMIPs (*p* ≤ 0.001) was found to be statistically significant (Figure 5d). This signifies that the binding of nanoMIPs is specific to the ERα positive cells.

**Figure 6:**
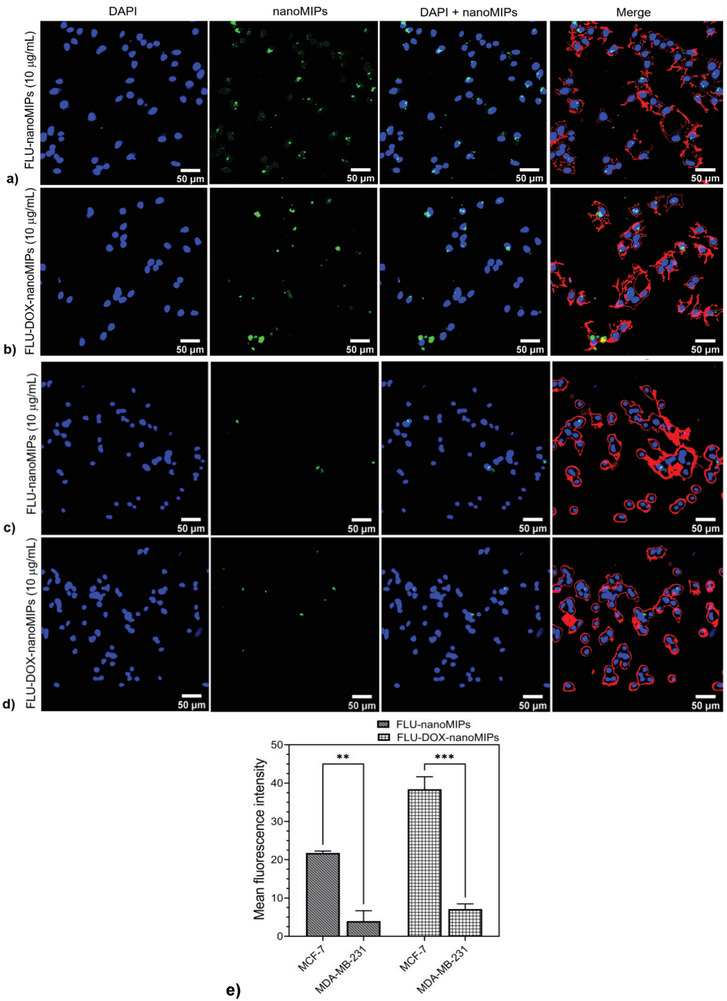
CLSM images (20×) for nanoMIPs incubated for 24 h at 37 °C a) MCF‐7 cells incubated with FLU‐nanoMIPs, b) MCF‐7 cells incubated with FLU‐DOX‐nanoMIPs, c) MDA‐MB‐231 cells incubated with FLU‐nanoMIPs, d) MDA‐MB‐231 cells incubated with FLU‐DOX‐nanoMIPs; Nucleus is stained with blue fluorescence (DAPI), nanoMIPs with green fluorescence, membrane with red fluorescence (WGA antibody Alexa Fluor 594), e) The mean fluorescence intensity of FLUnanoMIPs, FLU‐DOX‐nanoMIPs in MCF‐7 cells and MDA‐MB‐231 cells. ** *p* ≤ 0.01, *** *p* ≤ 0.001, versus MCF‐7.

The 12 h post‐incubation, specifically of FLU‐DOX‐nanoMIPs (illustrated in Figure S9, Supporting Information) and FLU‐nanoMIPs and FLU‐DOX‐nanoMIPs in both MCF‐7 and MDA‐MB‐231 cells after 24 h incubation (**Figure** **6**a‐d), was further examined. It was evident that FLU‐nanoMIPs and FLU‐DOX‐nanoMIPs (green) were able to cross the plasma membrane and get internalized into MCF‐7 cells (Figure 6c,d). On the contrary, no significant uptake of nanoMIPs in ERα‐negative cell line MDA‐MB‐231 was observed, as shown in Figure 6c,d. It has been reported that ERα binds with Caveolin‐1, which is one of the structural proteins of caveolae, and plays a major role in the trafficking of ERα to and from the cell surface and maintaining an environment for cell signaling and endocytosis of ERα.^[^ ^36^ ^]^ Thus, the internalization of nanoMIPs into MCF‐7 cells can likely be attributed to the ERα mediated caveolae‐dependent endocytosis.^[^ ^3^, ^37^ ^]^ Furthermore, the difference in MFI between MCF‐7 cells and MDA‐MB‐231 cells subjected to 10 µg mL^−1^ of FLU‐nanoMIPs (*p* ≤ 0.01) and FLU‐DOX‐nanoMIPs (*p* ≤ 0.001) was statistically significant (Figure 6e).

**Figure 7:**
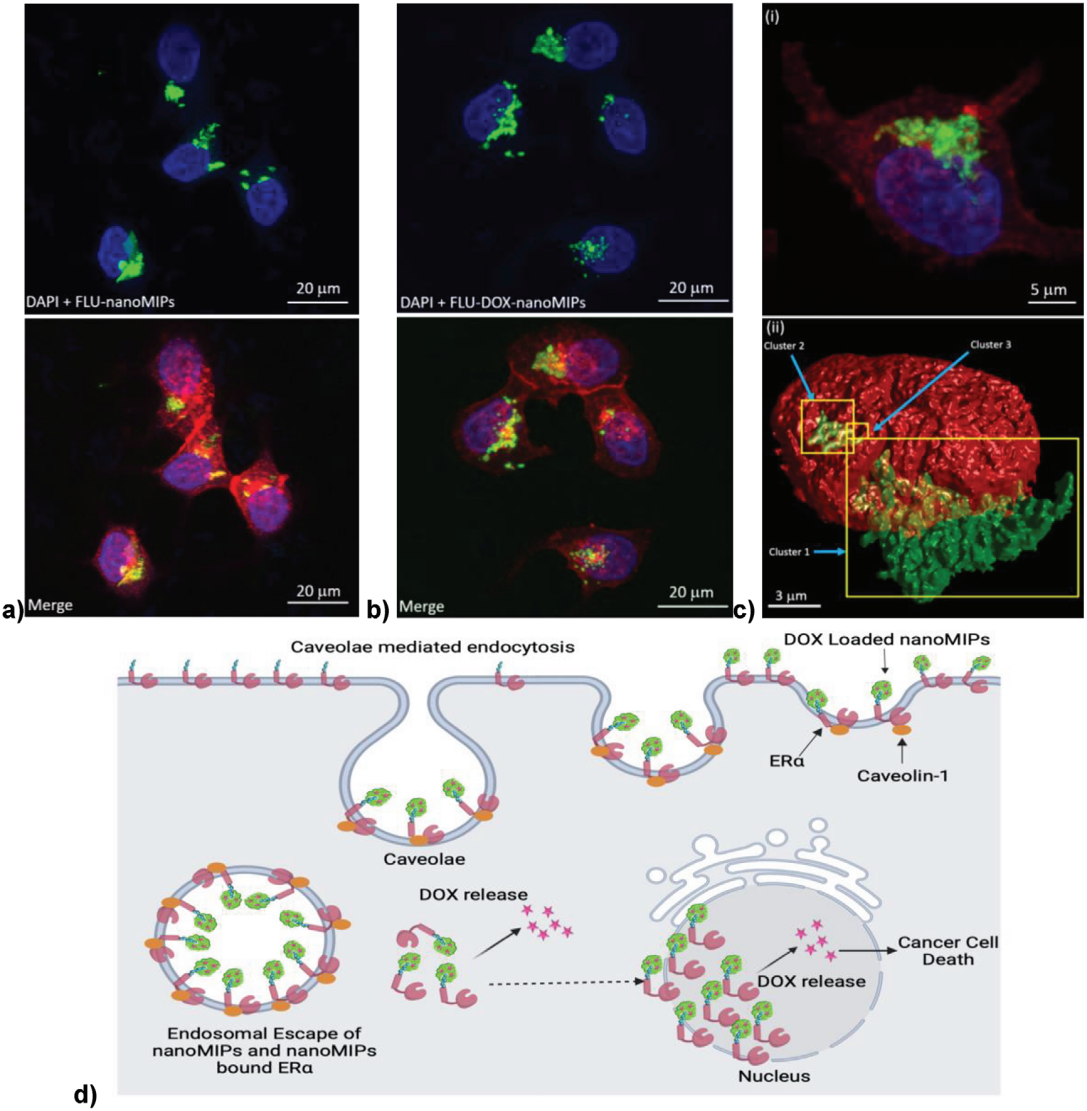
CLSM images (63×) of MCF‐7 cell line incubated with a) FLU‐nanoMIPs, b) FLU‐DOXnanoMIPs for 24 h at 37 °C, c) i) 3D image of FLU‐DOX‐nanoMIPs internalization in MCF‐7 cells ii) 3D render image showing the yellow areas (indicated by the blue arrows) are FLU‐DOX‐nanoMIPs (green) clusters colocalizing (hence, inside) the nucleus (red) of ERα positive MCF‐7 cells. The nucleus is stained with blue fluorescence (DAPI), nanoMIPs with green fluorescence, a membrane with red fluorescence (WGA antibody Alexa Fluor 594), d) Schematic representation of internalization of nanoMIPs through caveolae‐mediated endocytosis membrane ERα followed by translocation to the nucleus and release of DOX in the cytoplasm as well as in the nucleus.

After internalization (as illustrated at 63× in **Figure** **7**a for FLU‐nanoMIPs and Figure 7b for FLU‐DOX‐nanoMIPs), the nanoMIPs proceed to translocate into the nucleus, as demonstrated in Figure 7c (i). The 3D CLSM image confirmed the colocalization of the nanoMIPs within the nucleus of ERα positive cells, as evidenced by the overlapping fluorescence signal in yellow (Figure 7c (ii)). It has been observed that 3.53% of the volume of the nucleus (in the case of Figure 7c (ii)) colocalizes with FLU‐DOX‐nanoMIPs, furthermore, 15.61% and 1.71% of the volume of Cluster 1 and Cluster 2 respectively colocalized with the nucleus.

The ability of DOX‐loaded nanoMIPs to specifically bind to ERα positive MCF‐7 and its nucleus led to the release of DOX to both cytoplasm and nucleus, thus resulting in higher cytotoxicity compared to MDA‐MB‐231 cells treated with nanoMIPs. These findings were consistent with our cytotoxicity results observed in the MTT assay even at a lower concentration of DOX (≈2 µg) in the presence of 10 µg mL^−1^ nanoMIPs.

### 2.6 Evaluation of NanoMIPs in 3D Cancer Models

**Figure 8:**
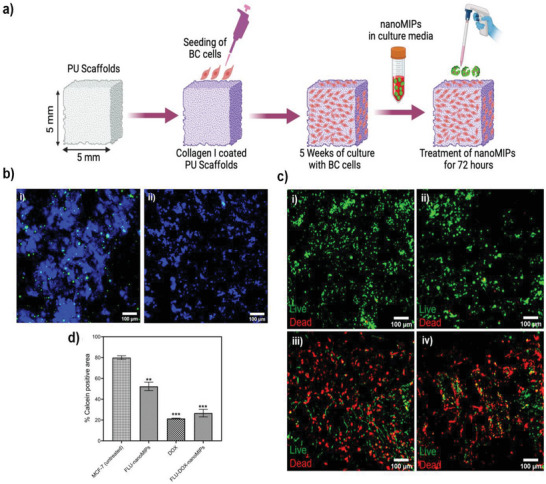
a) Schematic of cell culture seeding and treatment of nanoMIPs to 3D scaffold; b) CLSM images (10×) of 3D cancer models (poly‐urethane scaffolds coated with Collagen‐1) of MCF‐7 cell lines treated with i) FLU‐nanoMIPs, ii) FLU‐DOX‐nanoMIPs (after 72 h treatment) at 37 °C. The nucleus is stained with DAPI (Blue); nanoMIPs are in green, c) Cell viability (Live/dead staining) following nanoMIPs and DOX treatment; Representative images of scaffold sections for Live (green)/dead (red) staining after 3 days incubation of i) MCF‐7 control ii) FLU‐nanoMIPs iii) DOX drug iv) FLU‐DOXnanoMIPs, d) Quantification of Live (green) image area percentage (% calcein positive) using equivalent image analysis for i–iv). The analysis included multiple scaffolds (*n* = 3), various scaffold sections (*n* = 3), and a total of two images, with average mean values being showcased. The results represent the mean average values.

In order to investigate the action of nanoMIPs against MCF‐7 cells in a more biomimetic tissue environment, we conducted experiments in 3D porous polymer poly‐urethane (PU) scaffolds, surface modified with Collagen I for extracellular matrix (ECM) mimicry (**Figure** **8**a).^[^ ^18^, ^38^ ^]^ Collagen I is known to influence various factors related to the tumor microenvironment in BC, such as proliferation, survival, migration, and invasion. Changes in the structure of Collagen I have been observed during early BC development and are associated with local invasion. Additionally, the density of Collagen I has been found to correlate with the progression of breast cancer.^[^ ^39^ ^]^ Overall, the 3D cancer models are a better mimicry of essential aspects of tumor microenvironment (in vivo) in terms of structure, cell‐to‐cell and cell‐to‐extracellular matrix interactions, and spatial orientation, as compared to the simple 2D culture shown in the previous sections.^[^ ^18^, ^40^ ^]^

More specifically, as described in the methodology section, at week 5 of the 3D culture, the cells were exposed to various treatments for 72 h after which analysis of their spatial distribution and cell viability was performed. Our results showed that FLU‐nanoMIPs successfully penetrated the 3D cancer models of ERα positive MCF‐7 cell lines, as indicated by the green fluorescence in Figure 8b (i). Clear binding of FLU‐nanoMIPs with the nucleus (DAPI stained) has also been observed (co‐localization). These results demonstrated that these nanoMIPs can penetrate into complex 3D in vitro models. In the case of FLU‐DOX‐nanoMIPs (Figure 8b (ii)), binding to the 3D cancer models of ERα positive MCF‐7 cell lines was observed, but a smaller number of cells and lower fluorescence intensity were detected compared to FLU‐nanoMIPs. This suggests that after treatment with DOX‐loaded nanoMIPs, the cells that have up‐taken these nanoMIPs underwent cell death, detached from the scaffolds, and were eventually washed away during the washing process. Moreover, the cell viability of MCF‐7 cells in 3D cancer models was determined 72 h post‐treatment with nanoMIPs, DOX alone, and DOX‐loaded nanoMIPs. Figure 8c,d demonstrated the confocal images of spatial assessment and quantification of the live/dead staining of multiple sections of different 3D cancer models. It was evidenced from Figure 8c (i) that MCF‐7 cells were able to proliferate in 3D polyurethane‐based scaffolds as can be seen from the maximum % of calcein positive (green) area (80 ± 1.82%) in Figure 8d. However, lower cell viability of MCF‐7 cells (52.3 ± 4.0%) in 3D cancer models has been observed post‐FLU‐nanoMIPs treatment (72 h) at 10 µg mL^−1^ as compared to untreated BC MCF‐7 cells (Figure 8c (ii),d). This effect might be attributed to their selective binding to the helix 12 (H12) region of the ER receptor, a region pivotal for dimerization and transcriptional activation. This binding could elucidate the cytotoxicity of MCF‐7 cells.^[^ ^33c^ ^]^ Furthermore, the cell viability decreased more significantly after treatment with DOX alone (21.4 ± 0.44%) and DOX‐loaded nanoMIPs (26.6 ± 3.60%) at 10 µg mL^−1^ compared to MCF‐7 control cells (Figure 8c,d) and was evident from the increased red area (representing dead cells) in Figure 8c (iii) and civ for DOX and DOX loaded nanoMIPs respectively. These 3D scaffold findings were in good agreement with the cell viability results obtained from the MCF‐7 (2D) cell line and confer that these nanoMIPs can act as suitable drug carriers that exhibit targeted drug delivery and enhanced therapeutic activity.

3D cancer models enable a preliminary estimation of the penetration and anticancer activity of DOX‐loaded nanoMIPs and provide a better tumor‐mimicking environment compared to traditional 2D cell lines. Nonetheless, the ADME and toxicity of these materials cannot be addressed using single cellular 3D cancer models. This needs more advanced scaffolds or multi‐physiological systems with multiple organs such as the liver, kidney, heart, and many more to get more information about biodistribution, ADME, and toxicity.^[^ ^20^ ^]^ One can also consider xenograft animal models; however, these animal models do not usually possess a complete immune system. These nanoMIPs have been synthesized using a non‐degradable crosslinker, and this is a proof of concept study which showed that these double imprinted nanoMIPs can effectively act as a drug delivery vehicle for ER‐positive BC and also achieve nuclear delivery of anticancer drugs. It is worth mentioning that these nanoMIP compositions are expected to be excreted through the kidney, and these are not toxic to other organs as supported by previous in vivo studies.^[^ ^41^ ^]^ In future studies, we are considering enzyme‐cleavable crosslinkers (such as N,N'‐bis(acryloyl)cystamine) which will enable the synthesis of biodegradable nanoMIPs with further optimization and standardization. The scope of this work is the development and double imprinted nanoMIPs and their assessment via in vitro studies and 3D cancer models; however, for further ADME studies, advanced MPS (animal‐free models) or xenograft animal studies might be considered for further evaluation of these advanced nanomaterials.

## 3 Conclusion

A modified solid‐phase methodology was used to produce double imprinted nanoMIPs, which enabled the formation of two types of binding sites. The primary site exhibited high specificity toward ER and the secondary site toward DOX, a chemotherapeutic drug, enabling the use of these nanoparticles as targeted drug delivery carriers. The synthesized DOX‐loaded nanoMIPs had a spherical morphology with a typical size ranging from 140–170 nm. These materials rival the affinity of commercial antibodies, with a KD of 10 nm for ERα receptors as determined by SPR measurements. Moreover, these nanoMIPs specifically bound and elicited cytotoxicity (≈80%) to ERα positive cancer cells compared to ERα negative cell lines (≈15%) via nuclear delivery of DOX. This suggested that these smart nanocarrier systems can minimize off‐target side effects while improving drug efficacy. Furthermore, it was observed that FLU‐DOX‐nanoMIPs not only penetrated effectively to 3D cancer models of ERα positive BC cell lines but also elicited cytotoxicity, as witnessed by live‐dead staining. These findings provide strong evidence that these double‐imprinted nanoMIPs exhibit similar behavior in both 2D cell lines and complex 3D cancer models that closely resemble the tumor microenvironment. It is worth noting that the membrane‐to‐nuclear drug delivery behavior observed in this work is entirely novel and has not been reported on with molecularly imprinted or other hybrid nanoparticle systems. Importantly, ERα is also highly expressed in other types of cancer, such as endometrial, prostate, and ovarian cancer. Given the versatility of nanoMIPs, it is straightforward to extend this technology to the treatment of other cancers or other drug compounds. However, further studies are needed to explore the potential of these nanoparticles in clinical settings, their in vivo biodistribution and biocompatibility, and optimize their efficacy and safety. Overall, the use of targeted drug delivery systems such as nanoMIPs holds great promise for the future of cancer treatment due to their cost‐effective nature, robustness, high selectivity, and scalable production process.

## 4 Experimental Section

### Materials

Glass beads (53–106 µm diameter, Spheriglass 2429 CP00) were purchased from Blagden Chemicals (Kent, UK). *N*‐Isopropylacrylamide (NIPAM), *N,N′‐*Methylenebis(acrylamide) (Bis), N‐tert‐butylacrylamide (nTBA), *N‐*(3‐aminopropyl)methacrylamide hydrochloride (APMA), acrylic acid (AA), fluorescein O‐methacrylate, N,N,N′,N′‐tetraethylethylenediamine (TEMED), (3‐aminopropyl)trimethoxy‐silane (APTMS), doxorubicin HCl (DOX), dialysis cartridges (Vivaspin 20, 3 kDa molecular weight cutoff (MWCO) Polyether‐sulfone), Supelco polypropylene solid phase extraction tubes (60 mL) and 3‐[4, 5‐dimethylthiazol‐2‐yl]‐2,5‐diphenyltetrazolium bromide (MTT), 1‐ethyl‐3‐(3‐dimethylaminopropyl) carbodiimide (EDC), dipotassium phosphate, disodium phosphate, ethanolamine, *N*‐hydroxysuccinimide (NHS), and Tween 20 were purchased from Sigma–Aldrich, Poole, Dorset, UK. PierceTM Bicinchoninic Acid Assay (BCA) protein assay kit, ammonium persulfate (APS), methanol, acetone, acetonitrile, sodium hydroxide (NaOH), hydrochloric acid (33%, HCl), succinimidyl iodoacetate (SIA), potassium chloride, sodium acetate, sodium chloride, Oxoid phosphate‐buffered saline (PBS) tablets (Catalog number: BR0014G) and human recombinant ERα protein (Accession number NP_000116.2, Catalog number: A15674), Alexa Fluor 594 Wheat Germ Agglutinin (WGA) antibody, a Live/Dead Viability/Cytotoxicity Kit (Molecular Probes), DAPI, Invitrogen ERα primary monoclonal antibody (MA1‐310), Superclonal secondary antibody (Goat anti‐Mouse IgG Alexa Fluor 488) were purchased from Fisher Scientific UK Ltd (Loughborough, UK). CSHSLQKYYITGEAEGFPATV epitope of ERα was synthesized by Elabscience and obtained through Caltag Medsystems (Buckingham, UK). All chemicals and solvents were high‐performance liquid chromatography (HPLC) analytical grade and were used without any further purification. PBS solutions were prepared with deionized (DI) water with a resistivity of ≥18.2 MΩ cm.

### Preparation of Epitope Derivatized Glass Beads

60 g of glass beads (53–106 µm) were activated by boiling in 2 m NaOH (24 mL) for 15 min, then washed with double‐distilled water (ten times with 100 mL) until the pH of the washed solution was ≈7.4. Afterward, the glass beads were rinsed twice with acetone (100 mL) and dried at 80 °C for 2 h. Subsequently, the activated glass beads were incubated in a 24 mL solution of 2%, v/v APTMS in anhydrous toluene for 12 h for the silanization step. After the incubation step, the glass beads were transferred into a sintered funnel and washed with acetone (8 × 50 mL) and methanol (3 × 50 mL), and subsequently dried under vacuum. Then, 60 g of glass beads were placed in a solution of SIA (0.2 mg mL^−1^ in acetonitrile) for 2 h in the dark (0.4 mL solution/g glass beads). Afterward, the beads were washed with 400 mL of acetonitrile in a sintered glass funnel and incubated with 7 mg of cysteine‐modified peptide epitope (primary template) in 40 mL of deoxygenated 1× phosphate‐buffered saline (PBS) containing 5 mm EDTA, pH 8.2. Covalent imimmobilization of the epitope on the silanized bead was confirmed by performing a BCA assay.^[^ ^42^ ^]^

### Synthesis of NanoMIPs/NIPs Against ERα

The synthesis protocol was adapted from previous reports of Canfarotta and colleagues.^[^ ^13b^ ^]^ A monomer mixture containing NIPAM (39 mg), Bis (2 mg), nTBA (33 mg, pre‐dissolved in ethanol 1 mL), APMA (5.8 mg), AA (2.2 µL), and Fluorescein O‐methacrylate (FLU, 2.6 mg) was dissolved in 100 mL of PBS (5 mm, pH = 7.4). To fabricate DOX‐loaded doubly molecularly imprinted nanoparticles (DOX‐nanoMIPs) and fluorescein‐tagged DOX‐nanoMIPs (FLU‐DOX‐nanoMIPs), 3 mg of DOX was added to the monomer mixture. Fluorescein‐tagged nanoMIPs without DOX (unloaded) are named as FLU‐nanoMIPs. Non‐imprinted nanoparticles (NIPs) were produced according to the aforementioned method, except for the substitution of epitope‐derivatized beads with silanized beads. Briefly, the solution containing the monomer mixture was degassed under a vacuum sonicated for 10 min, and purged with N_2_ for 30 min.

Then, 60 g of ERα epitope derivatized glass beads were added to the solution under continuous N_2_ purging, and polymerization was initiated by adding a mixture containing 800 µL of APS aqueous solution (60 mg mL^−1^) and 24 µL of TEMED. The mixture was kept for 4 h at room temperature (RT) for polymerization, and afterward, poured into a solid‐phase extraction (SPE) cartridge (60 mL) fitted with a frit (20 µm porosity). The removal of low‐affinity nanoMIPs, polymer, and unreacted monomers was achieved by washing with distilled water (9 × 20 mL) at RT. Following that, 20 mL of distilled water pre‐warmed at 65 °C was poured into the SPE and placed in a water bath at 65 °C for 15 min. This step was repeated five times until ≈100 mL of high‐affinity nanoMIPs solution were collected. Concentrated nanoMIPs were obtained by evaporating the dispersion solvent in an oven for 24 h at 60 °C. The obtained nanoMIPs were further purified using a centrifugal dialysis cartridge fitted with a membrane of 3 kDa MWCO. Five washes with deionized water (10 mL) were performed and obtained nanoMIPs were re‐suspended in 50 mL of deionized water.

### Determination of DOX Loading and Release

A stock solution of DOX (100 µg mL^−1^) was prepared in water and further diluted in deionized water to the concentration of 1, 2.5, 5, 7.5, and 10 µg mL^−1^. UV–vis spectra were recorded between 200 and 600 nm using a Jenway 7200 UV–vis scanning spectrophotometer. The DOX calibration curve was plotted using absorbance (λ_max_ 254 nm) versus concentration (Figure S2, Supporting Information), and the linear calibration curve equation (*y = mx+b)* was used to estimate the DOX within the nanoMIPs. The calibration curve was plotted in the UV region at 254 nm because in the visible region, the fluorescein‐o‐methacrylate peak overlaps with the visible region peaks of DOX. The percent drug loading capacity (DLC) was calculated by taking the ratio of the amount of DOX encapsulated and the weight of nanoMIPs.

(1) $$\begin{array}{ccc} DLC\left(\%\right) & & =Encapsulated\;DOX\;/\\ & & Total\;weight\;of\;nanoMIPs\;or\;NIPs\times 100 \end{array}$$

The drug loading efficiency of DOX loaded into nanoMIPs was calculated with the following formula.

(2) $$\begin{array}{ccc} Drug\;loading\;efficiency & & =Encapsulated\;DOX\;in\;nanoMIPs\;/\\ & & amount\;of\;DOX\;fed\;initially\times 100 \end{array}$$

DOX‐loaded nanoMIPs and NIPs (2 mL each, polymer concentration was 1 mg mL^−1^) were poured into a dialysis bag (Spectrum Spectra/Por dialysis membrane with MWCO 12–14 kDa), and subsequently placed in a 8 mL release medium of phosphate buffer saline (PBS, pH 7.4). The dialysis system was kept at a temperature of 37 ± 0.2 °C with constant stirring at 120 rpm. Aliquots (0.5 mL) were taken from the release medium at predetermined time intervals and replenished with the same volume of fresh release medium. The concentration of DOX release was estimated using a UV–vis spectrophotometer, using a respective calibration curve of Figure S2 (Supporting Information). The cumulative % release was calculated using the following equation:

(3) $$\begin{array}{ccc} Cumulative\%release & & =\text{amount}\;\text{of}\;\text{DOX}\;\text{present}\;\text{in}\;\text{releasemedium}\;/\\ & & \text{Total}\;\text{encapsulatedDOX}\;\text{in}\;\text{nanoMIPs}\times 100 \end{array}$$

### Dynamic Light Scattering (DLS) and Electrophoretic Light Scattering Measurements

Dynamic light scattering (DLS) experiments were performed using Malvern Zetasizer Nano ZS to measure the hydrodynamic diameter (*D_h_*) of different nanoMIPs at 25 ± 0.1 °C. The instrument used a scattering angle of 173° and a laser wavelength of 632.8 nm. The size was measured at different times to evaluate the stability of the systems at 25 ± 0.1 °C. The dispersion of nanoMIPs in distilled water was subjected to sonication for 30 min and subsequently examined by DLS inside a 3 mL disposable polystyrene cuvette. In vitro stability of nanoMIPs was performed by mixing nanoMIPs with DMEM (GIBCO) low glucose media consisting of 10% foetal bovine serum (FBS, GIBCO) at 1:1 and incubated for different time intervals (0. 0.5, 4, 8, 12, and 24 h) at 37 °C. The stability of nanoMIPs was determined with the same instrument employed for DLS measurements.

### Morphology of NanoMIPs

For transmission electron microscopy (TEM), a nanoMIP solution (40 µg mL^−1^) was drop‐casted on the copper grids and images were captured using Hitachi HT7800 120 kV TEM machine (Tokyo, Japan) equipped with EMSIS CMOS Xarosa camera. For scanning electron microscopy (SEM) analysis, nanoMIP solutions were drop cast and dried on the glass chips (1 × 1 cm) and measurements were performed using a Tescan Vega 3LMU (Kohoutovice Czech Republic) machine with tungsten filament.

### Immobilization of NanoMIPs onto the SPR Sensor Surface

Carboxymethyl dextran hydrogel‐coated Au chips, purchased from Reichert Technologies (Buffalo, USA) were installed onto a Reichert 2 SPR following the manufacturer's instructions. The sensor surface was then preconditioned by running buffer PBST (PBS pH 7.4 and 0.01% Tween 20) at 10 µL min^−1^ until a stable baseline was obtained. The flow rate of 10 µL was maintained throughout the immobilization process. To activate carboxyl groups on the surface of the sensor chip, a freshly prepared aqueous solution (1 mL) of EDC (40 mg) and NHS (10 mg) was injected onto the sensor chip surface for 6 min.

To the activated surface, 300 µg of the nanoMIPs dissolved in 1 mL of the running buffer (PBST) and 10 mm sodium acetate were injected only to the left channel of the surface for 1 min. Finally, a quenching solution (1 m ethanolamine, pH 8.5) was injected for 8 min to deactivate carboxyl groups and wash away the unbound nanoMIPs. A continuous flow of running buffer (PBST) at 10 µL min^−1^ was maintained after nanoMIPs immobilization. SPR measurements were carried out after a stable baseline was achieved. The left channel was the working channel, and the right channel was the reference.

### Kinetic Analysis Using SPR

Kinetic analysis was initiated by injection of the running buffer PBST (blank) onto the nanoMIPs immobilized sensor surface for 2 min, followed by PBST for 5 min. The binding kinetics of individual nanoMIPs to the selected target were determined from serial dilutions (five concentrations in the 4–64 nm range) of the selected target under study. Each dilution was injected for 2 min (association) followed by PBST for 5 min (dissociation). After dissociation, the target was removed from the immobilized surface by injecting regeneration buffer (10 mm Glycine‐HCl, pH 2) for 1 min followed by PBST for 1 min. The same procedures were repeated for the remaining four dilutions of the target. After the analyses were completed, signals from the left channel were subtracted from signals from their respective reference channel (the right channel).

The SPR responses from five concentrations of the target compound (4–64 nm) were fitted to a 1:1 bio‐interaction (BI) model (Langmuir fit model) utilizing TraceDrawer software. Association rate constants (*k_on_*), dissociation rate constants (*k_off_*), and maximum binding (*B_max_*) were fitted globally, whereas the BI signal was fitted locally. The equilibrium dissociation constant (*K_D_*) was calculated from the ratio *k_off_/k_on_*.

### Cell Culture Conditions

MCF‐7 and MDA‐MB 231 were cultured in DMEM (GIBCO) low glucose media supplemented with 10% foetal bovine serum (FBS, GIBCO) and 1% penicillin/streptomycin (P/S). The cells were incubated in a humidified atmosphere of 5% CO_2_ and 95% air at 37 °C.

### In Vitro Cell Binding by Flow Cytometry

In vitro, cell binding of different nanoMIPs with cancer cells was determined using a BD LSRFortessa X‐20 flow cytometer. Cells were gently scraped, after washing with PBS, and resuspended in flow cytometry buffer (1×PBS, 0.5% BSA, 0.1% NaN_3_) at concentration 1 × 10^6^ cells in 100 µL. Prior to treatment, nanoMIPs were sonicated (10 min) and added to the cell suspension with the final concentration of 10 µg mL^−1^ and 40 µg mL^−1^. Following the 2‐h incubation, the cells were centrifuged, washed, and dispersed in a flow cytometry buffer, and subsequently, binding analysis was performed using a flow cytometer. Each experiment was done in triplicate. Data was analyzed using flowJo software, version 10.

### In Vitro Cytotoxicity Assessment

The MTT assay was used to evaluate the in vitro cytotoxicity of various nanoMIPs and pure DOX in MCF‐7 and MDA‐MB‐231 cells.^[^ ^43^ ^]^ For each experiment, 5000 cells per well were seeded into 96‐well plates and incubated for 24 h. The cells were then treated with the synthesized nanoMIPs and pure DOX at a concentration of 10 µg mL^−1^ for 24, 48, and 72 h. 2.5 mg of MTT was dissolved in 500 µL of phosphate‐buffered saline (PBS) and diluted to 5 mL with serum‐free DMEM medium. 200 µL of the MTT solution was added to each well of the plates after 24, 48, and 72 h of nanoMIPs treatment respectively, and the plate wrapped in aluminum foil were incubated at 37 °C for 4 h. After that, the media containing unbound MTT and dead cells was removed from each well, and 200 µL of DMSO was added to each well. The plates were shaken on an orbital shaker at 70 rpm for 30 min, and the absorbance was measured at dual wavelengths of 550 and 630 nm using a Spectramax M5^e^ (Molecular Devices, CA) multi‐mode automated microplate reader. The results were expressed as percentage cell viability, assuming the viability of control cells as 100%. Three independent experiments were performed for each study, and all measurements were performed in triplicate.

### Confocal Laser Scanning Microscopy (CLSM) Imaging

The nanoMIPs binding as well as cellular uptake in MCF‐7 and MDA‐MB‐231 cell lines was assessed at different time points (1 and 24 h). The cells were seeded at 20 000 cells per well (300 µL) in µ‐Slide 8 Well high ibiTreat chamber slides (Thistle Scientific, Uddingston, Glasgow, UK) and kept for 24 h to achieve 70–80% confluency. Prior to the treatment, nanoMIPs were washed with ethanol (70%) and then with PBS (two times) before adding to the cells, using centrifugation cartridges.

After 24 h, the medium was pipetted out and the nanoMIPs suspension (10 µg mL^−1^, in full media) was added to the chamber plates; then the plates were incubated for 1 or 24 h. Following the treatment, the chamber plates were washed three times with PBS (pre‐warmed) to remove the unbounded nanoMIPs. Afterward, the treated cells were fixed using 4% PFA solution prepared in PBS for 20 min at room temperature. PFA fixed chamber wells were then incubated with wheat germ agglutinin (WGA) with Alexa Fluor 594 (catalog no. W11262) for 10 min (dilution 1:200) to stain the plasma membrane. After washing the chamber wells with PBS (three times, 10 min each), DAPI (1:200 in PBS) was added for 20 min followed by the washing with PBS (three times). For the control experiment, MCF‐7 and MDA‐MB‐231 cells were fixed with 10% formalin incubated for 15 min at room temperature and permeabilized with Triton X‐100 (0.1% in PBS). The cells were blocked with 5% goat serum (in PBS) and stained overnight with 10 µg mL^−1^ of ERα primary antibody (MA1‐310 in 2.5% goat serum in PBS) at 4 °C. Afterward, the samples were washed twice with 1 mL of PBS, incubated with Alexa Fluor 488 secondary antibody (1:1000 in 2.5% goat serum in PBS) for 30 min, stained with 1 mL DAPI (1:10000 in PBS) for 5 min and finally the slides were mounted with media.

 The images were taken with a confocal laser scanning microscope Zeiss 880 inverted confocal microscope (Zeiss Europe) and Leica TCS SP8 STED 3× (Leica microsystems, Germany) using the following lasers i) 405 nm for DAPI (blue), ii) 488 nm for fluorescein tagged nanoMIPs and Alexa Fluor 488 secondary antibody (green) and iii) 561 nm for Alexa Fluor 594 (red). The images were analyzed using Image J software (Wayne Rasband, NIH, Bethesda, MD, USA).

### 3D Scaffold Preparation and Cell Culture

The thermal‐induced phase separation method was utilized to synthesize 3D porous polyurethane (PU)‐based scaffolds, which were then sterilized, and surface modified (coated) with Collagen I, as per the methodology described in.^[^ ^18^, ^19^, ^44^ ^]^

The MCF‐7 cell lines were seeded onto 3D cancer models measuring 5 × 5 × 5 mm^3^ at a seeding density of 0.5 × 10⁶ cells per scaffold. The cells were cultured for a duration of 5 weeks. Subsequently, incubated with various treatments, including FLU‐nanoMIPs, FLU‐DOX‐nanoMIPs, and DOX (10 µg mL^−1^) lasting 72 h, and the treatment was removed. After treatment, the scaffolds were characterized through sectioning, staining, and image analysis via CLSM.

### Spatial Evaluation of Live and Dead Cells via Imaging

To assess the spatial distribution of live and dead cells before and after treatment, model‐specific methods were employed. 3D cancer models were obtained at appropriate time intervals, snap‐frozen in liquid nitrogen (15 min), and subsequently, stored at −80 °C for further analysis. This preservation technique, widely utilized in tissue engineering, ensures sample integrity without compromising cell viability.^[^ ^18^, ^19^, ^45^ ^]^ These scaffolds were sectioned and washed two times with PBS prior to analysis. A Live/Dead Viability/Cytotoxicity Kit (Molecular Probes, Thermo Scientific, Loughborough, UK) was utilized for live/dead cell analysis. The sections of the 3D scaffold were stained with 2 µm of Calcein‐AM (from a 4 mm stock) and 4 µm of ethidium homodimer (from a 2 mm stock), and incubated for 1 h at 37 °C. The solution was subsequently removed, and the sections were washed twice with PBS and images were taken using a Zeiss 880 inverted confocal microscope (Zeiss Europe).

## Conflict of Interest

The authors declare no conflict of interest.

## Supporting information

Supporting Information

## Data Availability

The data that support the findings of this study are available in the supplementary material of this article.
