# Supplementary material for: Double Imprinted Nanoparticles for Sequential Membrane‐to‐Nuclear Drug Delivery
Source: Adv Sci (Weinh). 2024 Jul 8;11(36):2309976. doi: 10.1002/advs.202309976 (PMC11423068; doi:10.1002/advs.202309976)
Supplement: Supplementary file 1 — Supporting Information [file ADVS-11-2309976-s001.docx]

**Supporting Information**

**Double Imprinted Nanoparticles for Sequential Membrane-to-Nuclear Drug Delivery**

*Pankaj Singla^1,2,3 *^, Thomas Broughton^3,4,5^, Mark V. Sullivan^6^, Saweta Garg^1,2,3^, Rolando Berlinguer-Palmini ^7^, Priyanka Gupta^8^,* *Katie J Smith^3,4^, Ben Gardner^3,4^, Francesco Canfarotta^9^, Nicholas W. Turner^6^, Eirini Velliou^8^, Shoba Amarnath^3,4,5^ and Marloes Peeters^1,2,3^****^*^***

^1^ Department of Chemical Engineering, Engineering building A, East Booth Street, The University of Manchester, Oxford Road, M13 9PL

^2^ School of Engineering, Merz Court, Claremont Road, Newcastle University, Newcastle Upon Tyne, NE1 7RU, United Kingdom

^3^ Center for Cancer Research, NU Cancer, Faculty of Medical Sciences, Newcastle University, Newcastle Upon Tyne, NE2 4HH, United Kingdom

^4^ Immune Regulation Laboratory, NU Biosciences, Faculty of Medical Sciences, Newcastle University, Newcastle Upon Tyne, NE2 4HH, United Kingdom

^5^NIHR, Biomedical Research Centre, Newcastle University, Newcastle Upon Tyne, NE2 4HH, United Kingdom

^6^ Department of Chemistry, Dainton Building, University of Sheffield, Sheffield, S3 7HF, United Kingdom

^7^ The Bio-Imaging Unit, William Leech Building, Medical School, Newcastle University, Newcastle Upon Tyne, NE2 4HH, United Kingdom

^8^ Centre for 3D models of Health and Disease, Division of Surgery and Interventional Science, University College London, W1W 7TY, United Kingdom

^9^ MIP Discovery, The Exchange Building, Colworth Park, Sharnbrook, MK44 1LQ, Bedford, United Kingdom

**---------------------------------------------------------------------------------------------------------------------------------------*Corresponding authors, E-mail address:** Pankaj.singla@manchester.ac.uk (Pankaj Singla)**,** marloes.peeters@newcastle.ac.uk (Marloes Peeters)

| Batch | ERα epitope | NIPAM | nTBA | AA | APMA | Bis | FLU | DOX |
| --- | --- | --- | --- | --- | --- | --- | --- | --- |
| nanoMIPs | + | + | + | + | + | + | - | - |
| FLU-nanoMIPs | + | + | + | + | + | + | + | - |
| FLU-DOX-nanoMIPs | + | + | + | + | + | + | + | + |
| DOX-nanoMIPs | + | + | + | + | + | + | - | + |
| NIPs | - | + | + | + | + | + | - | - |
| FLU-NIPs | - | + | + | + | + | + | + | - |
| FLU-DOX-NIPs | - | + | + | + | + | + | + | + |

**Table S1.** Composition of different batches of the nanoMIPs and NIPs fabricated in this study.

**DLS measurement of control nanoMIPs and non-imprinted polymeric nanoparticles (NIPs):** The hydrodynamic diameter (*D_h_*) of nanoMIPs, NIPs, FLU-NIPs and FLU-DOX-NIPs were determined to be 110 ± 2 nm (PDI=0.112), 108 ± 3 nm (PDI=0.116), 121 ± 4 nm (PDI=0.110) and 143 ± 3 nm (PDI=0.131) respectively, as shown in **Table S2**. These results indicate that the loading of fluorescein-o-methacrylate and DOX within the NIPs led to the increased *D_h_* of FLU-NIPs and FLU-DOX-NIPs as compared to NIPs (control). Furthermore, PDI values of nanoMIPs and different NIPs were observed to be less than 0.2 suggesting homogeneity of the produced nanoparticles.

**Table S2.** Intensity weighted size (hydrodynamic diameter) of control nanoMIPs and NIPs

|  | Hydrodynamic diameter (*D_h_*), nm | Polydispersity index (PDI) |
| --- | --- | --- |
| nanoMIPs | 110 ± 2 | 0.112 |
| NIPs | 108 ± 3 | 0.116 |
| FLU-NIPs | 121 ± 4 | 0.110 |
| FLU-DOX-NIPs | 143 ± 3 | 0.131 |

**Table S3.** DOX loading, loading efficiency and loading capacity of different batches of DOX loaded nanoMIPs and NIPs.

|  | Loading DOX concentration (μg/100 μg) | Loading efficiency | Loading Capacity |
| --- | --- | --- | --- |
| DOX-nanoMIPs | 17.28 ± 0.1 | 57.6 ± 0.33 % | 17.28 ± 0.1 % |
| FLU-DOX-nanoMIPs | 19.33 ± 0.16 | 64.43 ± 0.53 % | 19.27 ± 0.16 % |
| FLU-DOX-NIPs | 18.37 ± 0.12 | 61.12 ± 0.40 % | 18.21 ± 0.12% |

**a)**
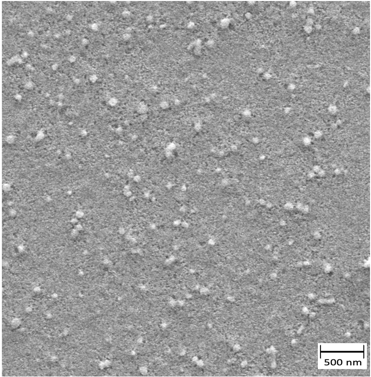
 **b)**
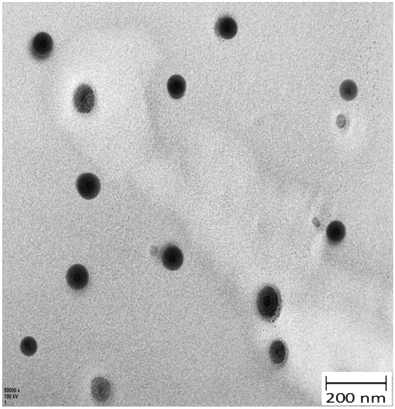


**Figure S1.** Characterization: **a)** representative SEM images of DOX-nanoMIPs; **b)** TEM image (25000x) DOX-nanoMIPs





**Figure S2.** Calibration curve of DOX absorbance (l_max_ 254 nm) *vs.* concentration (1, 2.5, 5, 7.5 and 10 μg/mL) obtained from UV-visible spectra.


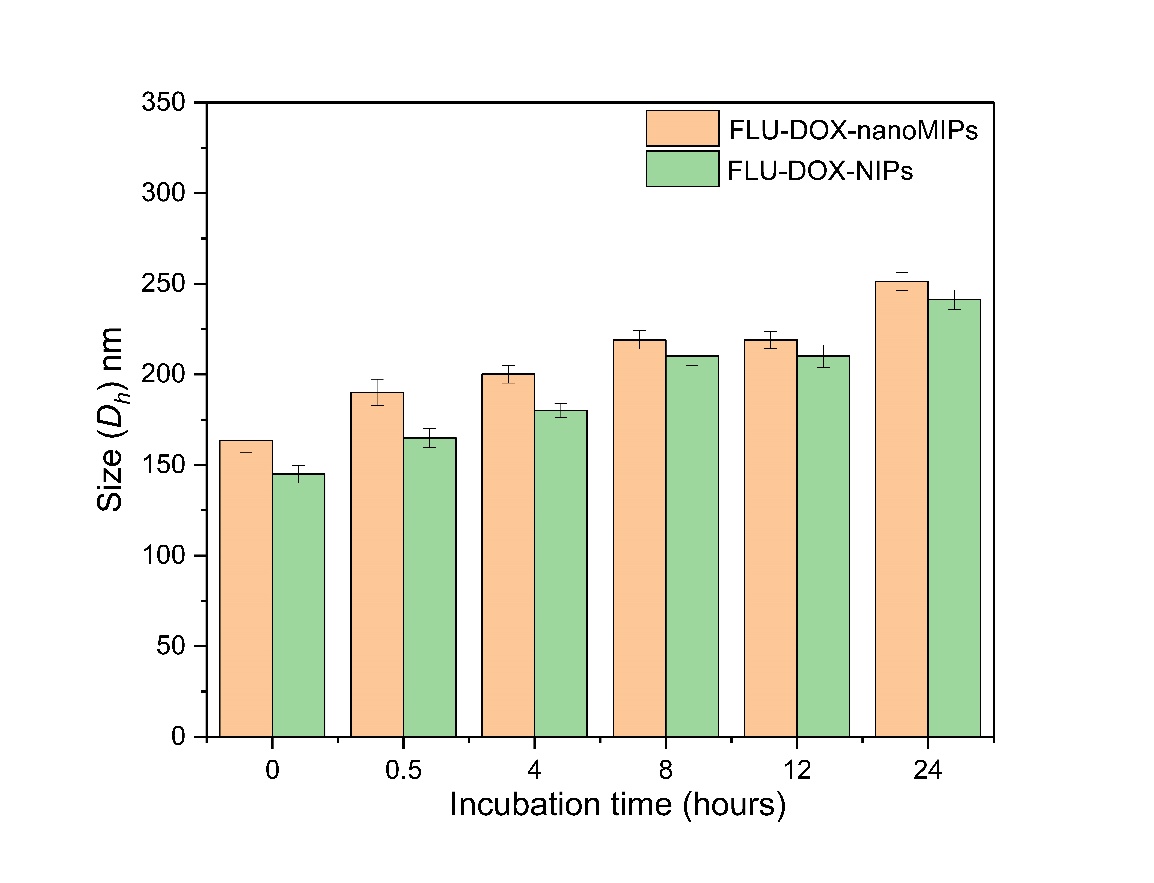


**Figure S3.** Time dependent stability of nanoMIPs (FLU-DOX-nanoMIPs and FLU-DOX-NIPs) in DMEM culture media.


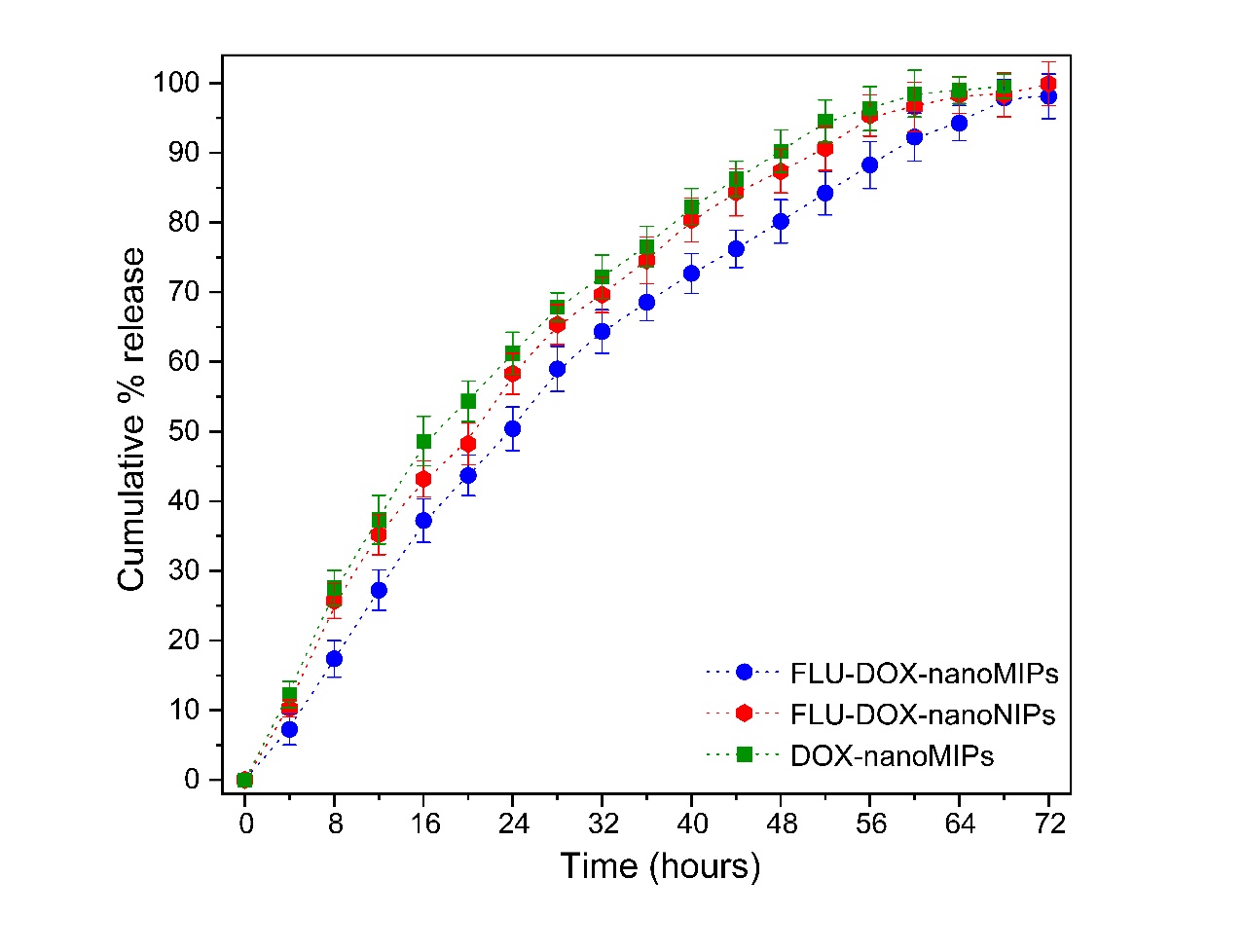


**Figure S4.** The cumulative % release of DOX by FLU-DOX-nanoMIPs, FLU-DOX-nanoNIPs and DOX-nanoMIPs in phosphate buffer saline (pH = 7.45). Data are shown as mean ± SD.


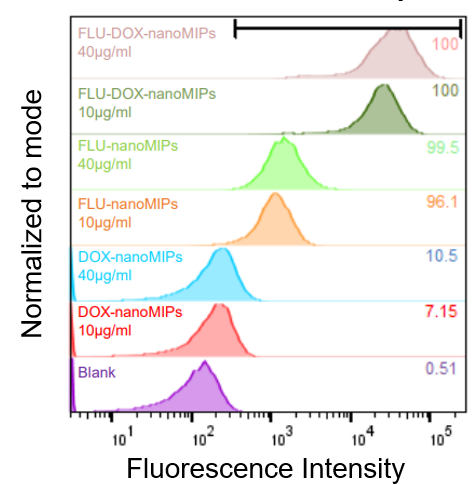


**Figure S5.** *In vitro* flow cytometry binding assay, MCF-7 cells were incubated with 10 μg/mL and 40 μg/mL of DOX-nanoMIPs, FLU-nanoMIPs and FLU-DOX-nanoMIPs.


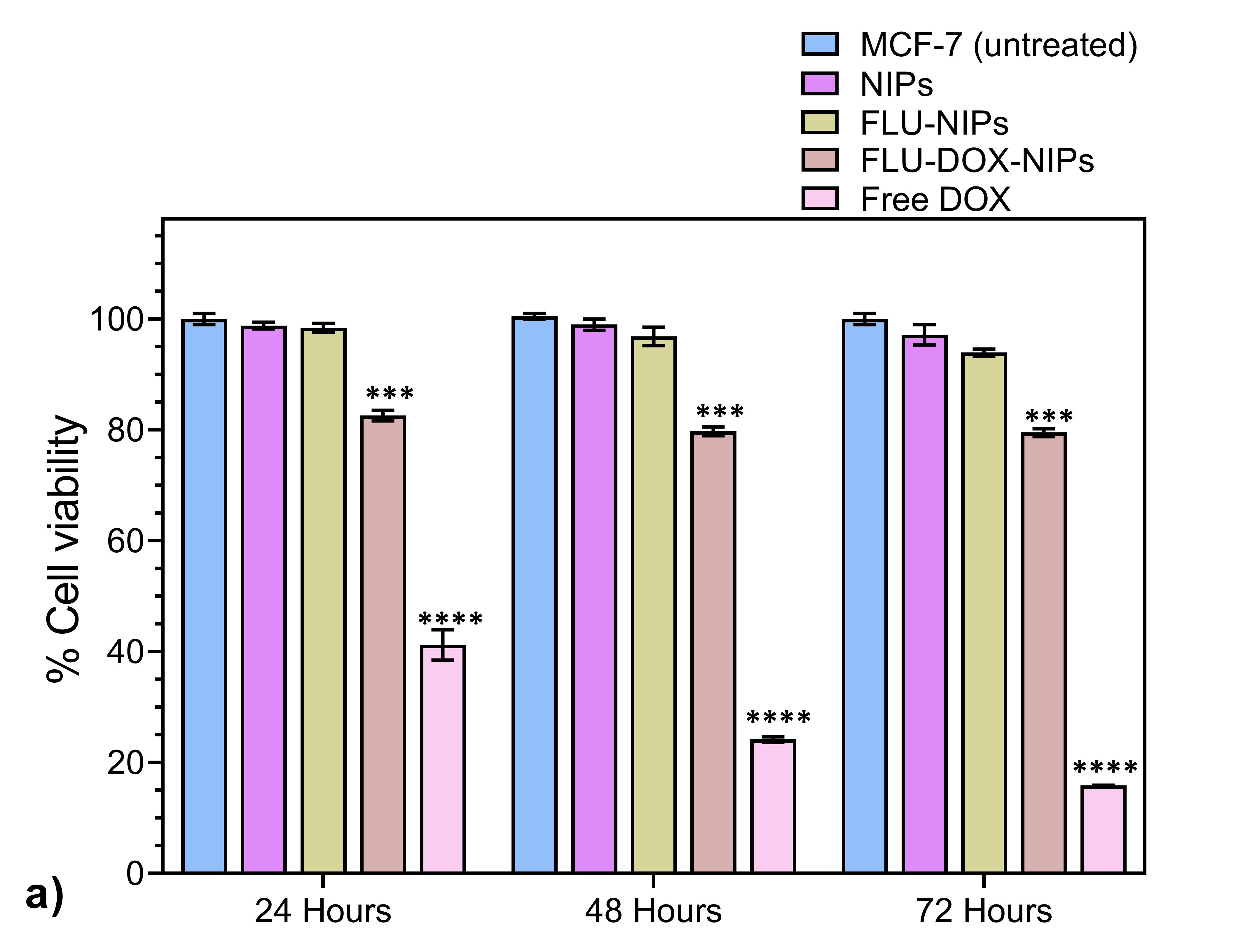

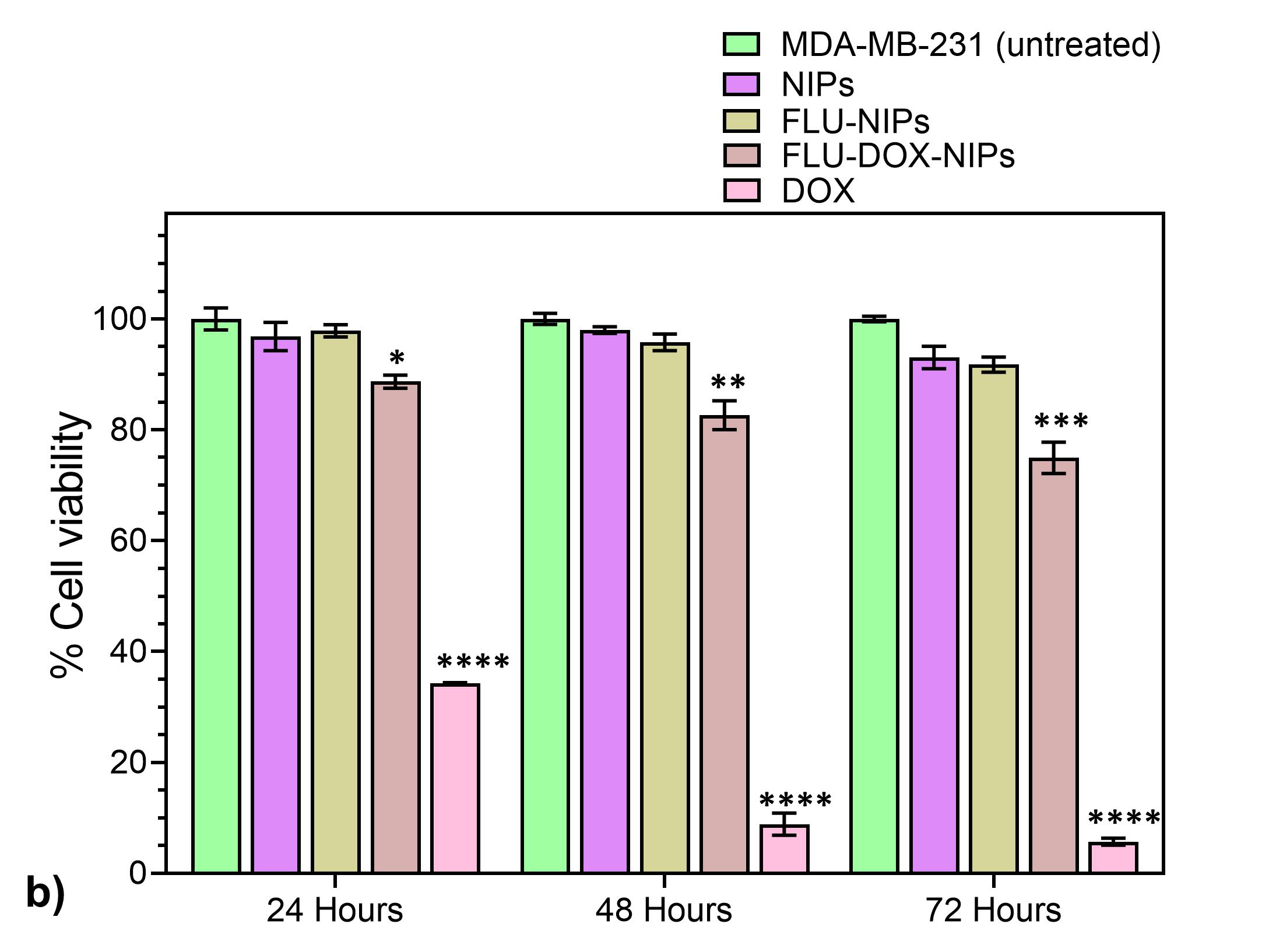


**Figure S6.** *In vitro* cell viability assay, at 10 μg/mL for each treatment with NIPs, FLU-NIPs, FLU-DOX-NIPs and Free DOX, **a)** MCF-7 and **b)** MDA-MB-231. Data is expressed as mean ± SEM of three measurements. *** P ≤ 0.001, **** P ≤ 0.0001 *vs.* MCF-7 control (**a**), *P ≤ 0.05, ** P ≤ 0.01, *** P ≤ 0.001, **** P ≤ 0.0001 *vs.* MDA-MB-231 control (**b**).


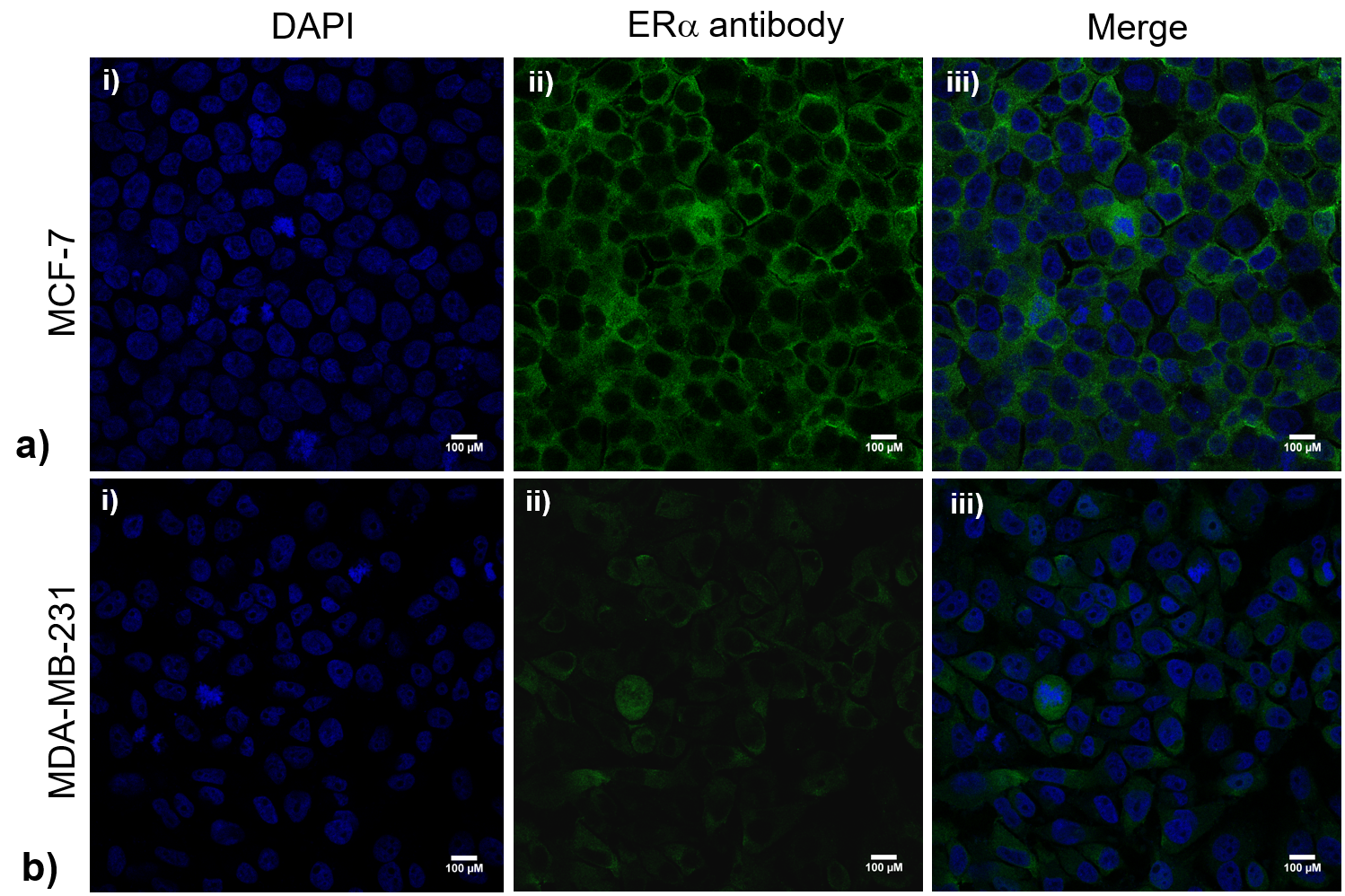


**c)**
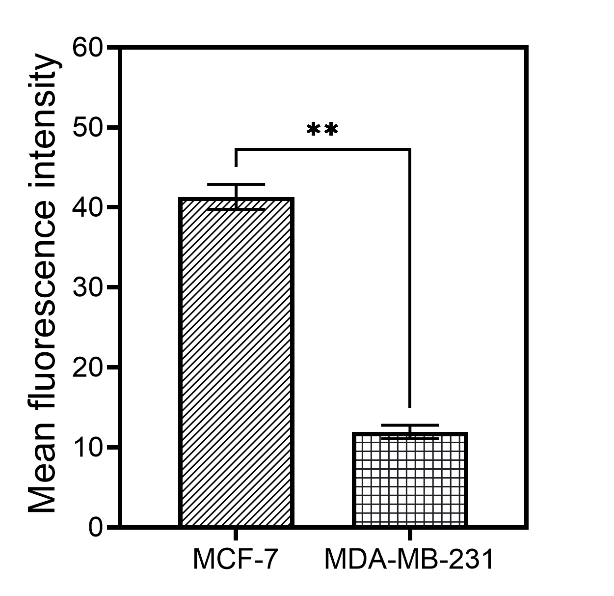


**Figure S7.** CLSM images (40X) for **a)** MCF-7, **b)** MDA-MB-231 stained with ERα primary antibody incubated overnight at 4°C, and subsequently stained with anti-mouse Alexa Fluor 488 secondary antibody for 30 min at room temperature and DAPI nuclear staining for 5 min at room temperature. **(i)** DAPI (blue), **(ii)** ERα antibody (green), **(iii)** merged, **c)** The mean fluorescence intensity of ERα antibody in MCF-7 cells and MDA-MB-231 cells. Data is expressed as the mean of three SEM measurements, ** P ≤ 0.01 *vs.* MCF-7 control.


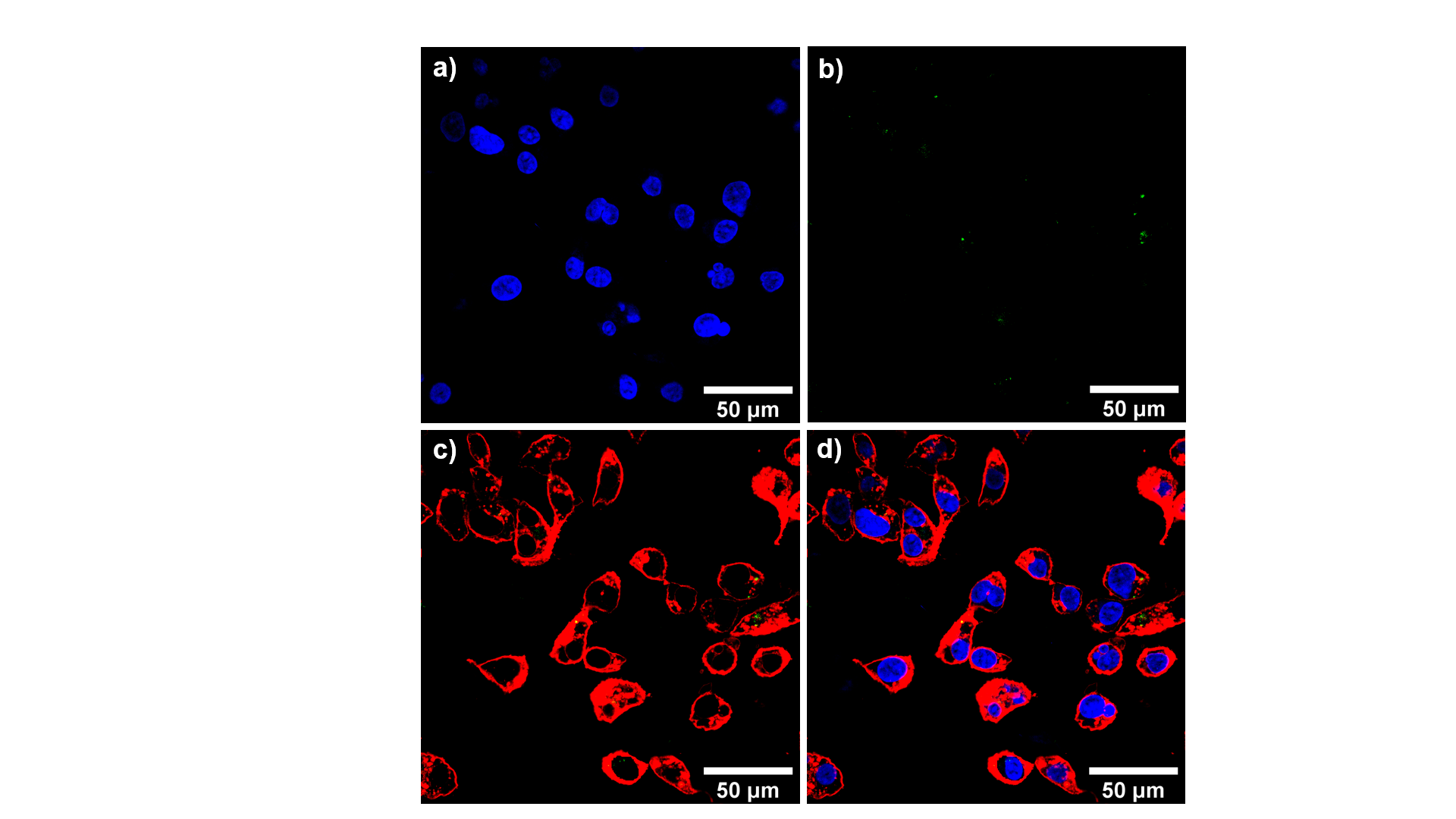


**Figure S8.** CLSM images (40X) for MDA-MB-231 incubated with FLU-DOX-nanoMIPs for 1 hour at 37 °C **(a)** DAPI, **(b)** FLU-DOX-nanoMIPs with green fluorescence, **(c)** plasma membrane with red fluorescence (WGA antibody Alexa Fluor™ 594) with FLU-DOX-nanoMIPs, **(d)** merged.


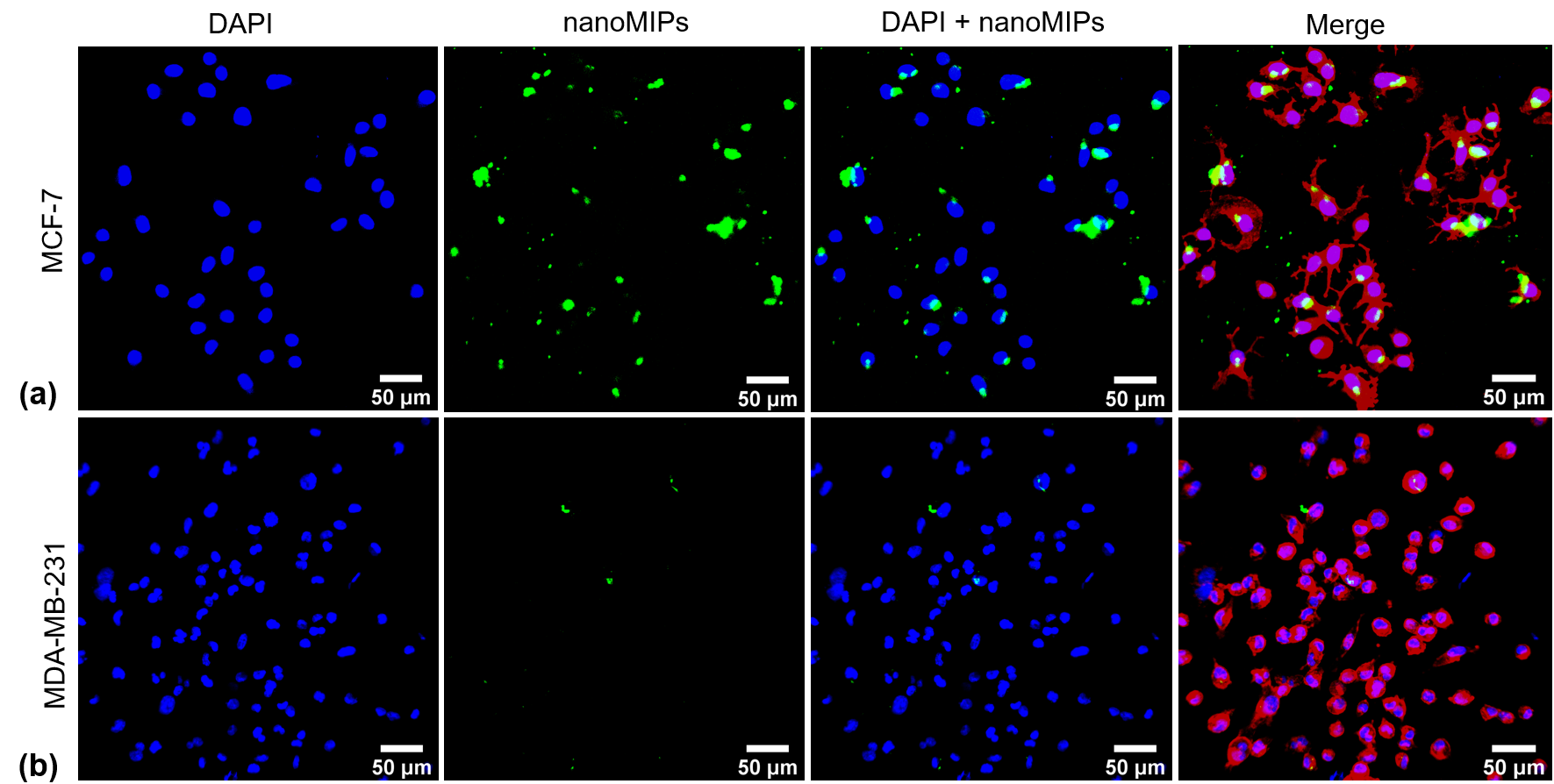


**Figure S9.** CLSM images (20X) for FLU-DOX-nanoMIPs incubated for 12 hours at 37 °C with **(a)** MCF-7 cells, **(b)** MDA-MB-231 cells. Nucleus is stained with blue fluorescence (DAPI), nanoMIPs with green fluorescence, plasma membrane with red fluorescence (WGA antibody Alexa Fluor™ 594).
